# Oral Immunization with HIV-1 Envelope SOSIP trimers elicits systemic immune responses and cross-reactive anti-V1V2 antibodies in non-human primates

**DOI:** 10.1371/journal.pone.0233577

**Published:** 2020-05-29

**Authors:** Bridget S. Fisher, Nicholas Dambrauskas, Olesya Trakhimets, Daniela V. Andrade, Jeremy Smedley, Donald L. Sodora, D. Noah Sather

**Affiliations:** 1 Seattle Children’s Research Institute, Seattle, WA, United States of America; 2 Washington National Primate Research Center, University of Washington, Seattle, WA, United States of America; 3 Department of Pediatrics, University of Washington, Seattle, WA, United States of America; 4 Department of Global Health, University of Washington, Seattle, WA, United States of America; Emory University School of Medicine, UNITED STATES

## Abstract

Development of a successful HIV vaccine is dependent upon a determination of the optimum antigen and adjuvant as well as choosing an optimal site for vaccine delivery. The site of delivery is particularly relevant as HIV transmission generally requires that the virus crosses a mucosal membrane to infect a new host. Here we undertake a pilot study comparing three vaccine delivery routes, two to the oral cavity (intraepithelial (iEp) and needle-free (NF-Injex)) as well as intramuscular (IM) delivery. These vaccinations utilized a recombinant HIV-1 Env trimer 10042.05 from an elite neutralizer, subject VC10042, that has previously induced high titers of cross-clade reactive V1V2 antibodies. The 10042.05.SOSIP fused trimer was administered with adjuvants R848 (Resiquimod), MPLA and Alhydrogel to characterize the innate cellular and anti-HIV Envelope (Env) antibody responses following the administration of the vaccine to the oral mucosa. Oral delivery of the 10042.05.SOSIP induced high titers of anti-V1V2 antibodies, which together with previous studies, indicates an immunogenic bias toward the V1V2 regions in 10042-derived Envs. Both types of oral vaccine delivery resulted in immunologic and serologic responses that were comparable to the IM delivery route. Furthermore, induction of anti-V1-V2 specific antibodies was best following iEp delivery of the oral vaccine identifying this as the optimal method to orally deliver this vaccine formulation.

## Introduction

The HIV-1 epidemic continues to exact a massive human and economic toll. Efforts to increase access to antiretroviral therapies have brought the number of yearly deaths from HIV-1 to below 1 million per year (UNAIDS). However, decreases in the rate of viral acquisition have not kept pace and remain at 1.8M new infections each year, pushing the total number of infections toward 37 million people worldwide. Thus, development of an effective vaccine remains the ultimate goal for the induction of a protective, long lasting memory and rapid recall immune response to prevent infection from a future HIV exposure.

An effective HIV-1 vaccine remains elusive, with only one clinical trial, RV144, resulting in efficacy against viral acquisition [1]. This trial resulted in ~34% reduction in viral acquisition, and follow-on analyses indicated that neutralizing antibodies were not associated with protection from infection [1–3]. Rather, anti-V1V2 loop IgG was identified as the major correlate of reduced risk. In addition, Fc-mediated functional activity of the anti-V1V2 antibodies are associated with reduced risk of infection [3–9]. These findings imply that non-neutralizing activities can contribute to protection in an eventual vaccine. While the field continues to focus on eliciting broadly neutralizing antibodies (bNAbs) [10–13], there remain significant hurdles to overcome, including a need to induce a high degree of somatic mutation and the lack of germline B cell receptor recognition by Env immunogens [14–18]. Therefore, optimizing strategies that elicit V1-V2 loop HIV antibody responses remains one that is achievable and desirable. Thus, there is significant need for the development vaccine antigens and delivery modalities that can accomplish this goal in a safe and effective manner.

There are currently licensed mucosally-administered vaccines for five different pathogens, most of which are administered orally to the gastro-intestinal associated lymphoid tissue (e.g., poliovirus) or nasally (e.g., influenza) [19, 20]. Such vaccines are known to elicit both local and systemic immune responses but can show variable efficacy, and some carry safety concerns due to their formulation. Vaccination in the intravaginal and intrarectal mucosal tissues can induce strong local immune responses, but systemic responses induced by these routes are modest [20, 21]. Direct vaccination into the oral mucosal tissue is a mucosal vaccination site that has been demonstrated to induce systemic immunity and has been shown to enhance immunity at distal mucosal sites [22–27]. The oral mucosa is rich with accessible lymphoid tissue, including the ringed arrangement of lymphoid tissue known as the Waldeyer’s tonsillar ring (WTR), which contains the tonsils, adenoids, and other lymphoid tissue [28–30]. Vaccination into the oral mucosal tissue may allow for the localized, rapid uptake of antigen into primary lymphoid tissues to form germinal centers and promote antibody maturation. Further, the oral mucosal subepithelial layers are enriched with Langerhans cells, lymphocytes, and macrophages that may be readily engaged by vaccination and may promote efficient antigen uptake and presentation, along with robust trafficking to primary lymphoid tissues. Two recent studies evaluated oral vaccination to induce HIV-1 immunity [24, 25]. Both studies utilized vectored vaccines combined with recombinant protein and found that oral mucosal immunization induced systemic antibody responses that were similar in magnitude to that of systemic immunization. However, the induction of immunity by oral or IM immunization appears to be qualitatively different, eliciting nuanced innate responses.

We previously evaluated a recombinant HIV-1 Env trimer 10042.05 derived from an elite neutralizer, subject VC10042, in rabbits and found that it induced high titers of cross-clade reactive V1V2 antibodies and low potency neutralizing antibodies [31–33]. Here, the recombinant Env was maintained as a native-like trimer by engrafting the C5-MPER region of BG505.SOSIP.559 native-like trimer and introducing disulfide bonds to increase the stability of the V3 loop [34–36], and ensured that this 10042.05.SOSIP binds to the trimer-specific antibody PGT151 [37]. The goal was to conduct a pilot study in non-human primates to evaluate the induction of immunity by native-like SOSIP trimers via oral vaccination in comparison to peripheral IM vaccination. We undertook immunizations at the oral mucosa by two approaches, intra-epithelial injection (iEp) and needle free (NF), as well as through IM injection. Innate immune responses were evaluated through transcriptomics and flow cytometry, in addition immune cell phenotypes (B cells) and anti-HIV serological responses to the vaccine were measured. Our findings indicate that oral vaccine delivery results in the initiation of the adaptive immune response with similarities to IM with regard to numerous immunologic and serologic responses. Importantly, the oral delivery route the 10042.05.SOSIP induced high titers of anti-V1V2 antibodies in NHP. Overall, our findings indicate that the iEp oral delivery route was superior to the needle-free approach with regard to the extent of the anti-V1-V2 antibody induction and that oral vaccination of Env trimers is a viable route for inducing immunity against HIV-1.

## Results

### Immunization of non-human primates

The fused trimeric 10042.05 Env previously tested was re-designed to express as a cleaved SOSIP trimer molecule. The gp120 region of 10042.05 was fused with the BG505 C5-gp41 sequence, as previously reported [35, 38], linked by a furin cleavage site (RRRRRR) and the standard disulfide linkages to tether the gp120 and gp41 subunits after cleavage (501C-605C). A disulfide bond (201C-433C) was introduced to stabilize the conformation of the V3 loop [34]. Other mutations known to stabilize trimerization were also introduced (see materials and methods) [36]. After a three-step production protocol consisting of affinity and size exclusion chromatography, native-like trimer fractions binding to the quaternary-specific bNAb PGT151 were pooled, QC’d by analytical SEC and blue native PAGE (S1 Fig), and analyzed for binding by Octet Bio Layer Interferometry (S2 Fig). The final trimer formulation bound to PGT151 and 8ANC195, two trimer-specific bNAbs, confirming native-like trimer formation (S2A Fig). The protein also bound to CD4-IgG2 (S2B Fig) and 447-52D (S2D Fig), but exhibited no binding to VRC01 and no binding to PG16 (S2C Fig). The lack of binding to VRC01 and PG16 is unsurprising, as the parental virus is completely resistant to neutralization by both bNAbs [33].

**Fig 1:**
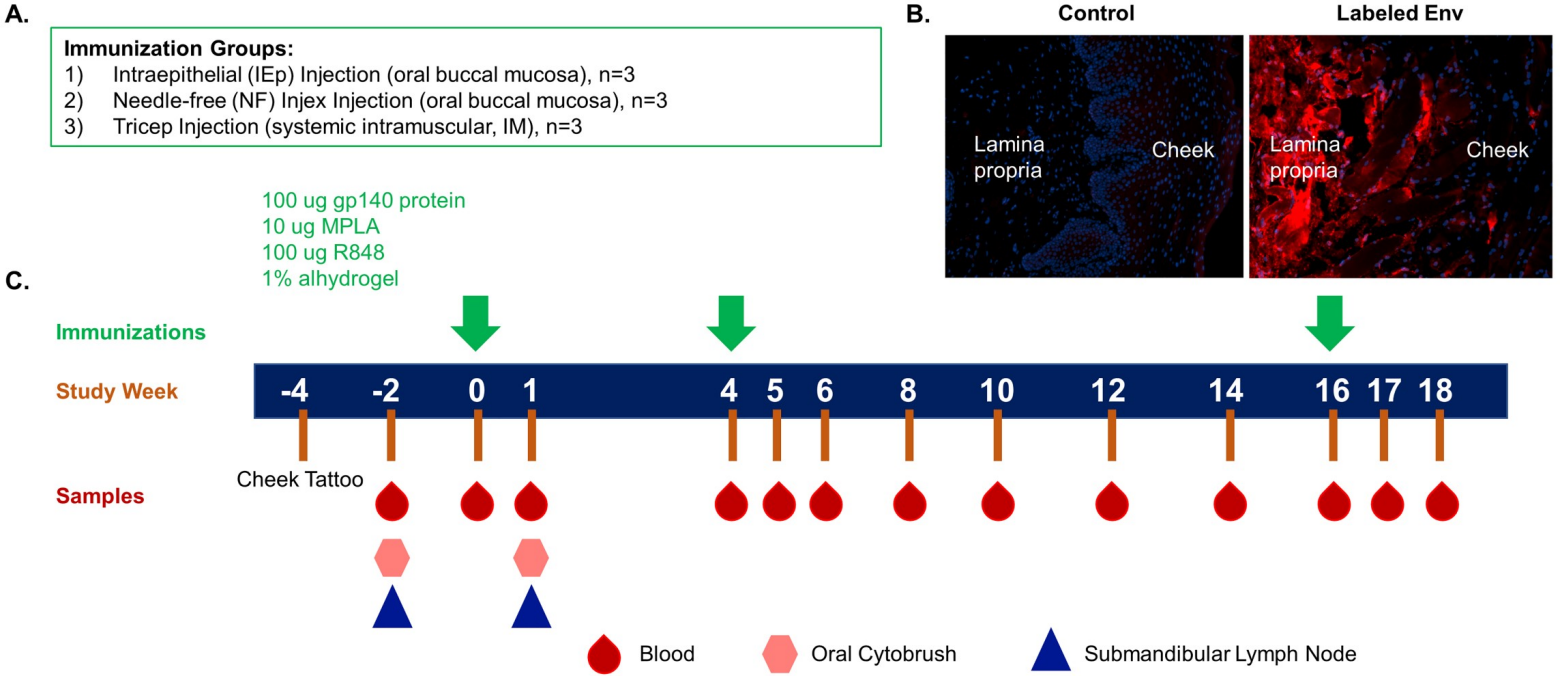
Study design and sampling timeline. (A) Rhesus macaques were assigned to immunization groups: oral buccal immunizations by a needle-free (NF, n = 3) delivery system or by a needle injection placed in the intraepithelial space (iEp, n = 3) or parenteral intramuscular (IM, n = 3) tricep injection group. (B) rhesus macaque cheek tissue was injected with 10042.05.SOSIP by needle free injection. Following injection the tissue was thin-sectioned and stained with mAb 447-52D. Env within the tissues exhibited red fluorescence, whereas no-Env controls had no mAb signal. (C) Immunizations were administered at study weeks 0, 4 and 16 with blood, oral cytobrushes and submandibular lymph nodes collected at the timepoints indicated for immunogenicity evaluation.

We conducted a pilot immunization study of three groups of rhesus macaques, each containing three animals, with the goal of comparing intramuscular peripheral immunization (IM) with two modes of oral vaccination, intra-epithelial (iEp) and needle free (NF). Although groups of three animals were sufficient for this exploratory pilot study, the small group size limited our statistical power to detect differences in the serological readouts. All groups received the same dose of 100 micrograms of 10042.05.SOSIP trimer. All vaccines were combined with R848 (Resiquimod), a TLR 7/8 agonist known to stimulate B cell maturation, monophosphoryl lipid A (MPLA), a TLR 4 agonist, and Alhydrogel (aluminum hydroxide) as adjuvants. IM doses were split and injected into each tricep. The dose and volume for NF and iEp were equivalent. Thus, all animals received the same amount of Env trimer per immunization, but the volume and absolute amount of Alhyrdogel differed between oral and IM routes. However, all immunizations contained the same vol/vol percentage of Alhydrogel, which was prepared according to the manufacturer’s instructions. All animals were immunized three times, at weeks 0, 4, and 16. The immunization, dosing regimen, and sampling schedule are shown in Fig 1.

All animals received a cheek tattoo at week -4 to enable a landmark that could be used for locating injections and tissue biopsies in those macaques that were orally vaccinated. For iEp injections, the concentrated vaccine was injected at a shallow angle into the intra-epithelial tissue with a fine gauge needle. For the NF arm, we used the Injex needleless injection system. This system uses compressed air to drive a jet of liquid that penetrates into the tissue and disperses, rather than creating a fluid body as is thought to occur with needle injection. This delivery method differs from that of Jones and colleagues [24], which uses sonication to drive the fluid injection into the tissue. Injex NF uses a mechanical device to create pressure behind a needle-free injection syringe. Upon actuation of the trigger the pressure moves the plunger, spraying liquid out of the nozzle and through a spacer. The jet of liquid penetrates the tissue as a spray and disperses [19, 39]. To evaluate the dispersal pattern of the Envs after Injex inoculation, we mock-vaccinated rhesus macaque cheek biopsy tissue. Following injection, the tissue was fixed, sectioned, and probed with fluorescently labeled 447-52D. We observed even dispersal of the Env across multiple layers of cheek tissue, indicating a broad and even distribution throughout the tissue (Fig 1B). In the NF arm, loaded Injex syringes with a spacer were placed lightly against the cheek tissue at a 90-degree angle and the payload delivered into the tissue.

### Oral immunization induces local transcriptomic changes

**Fig 2:**
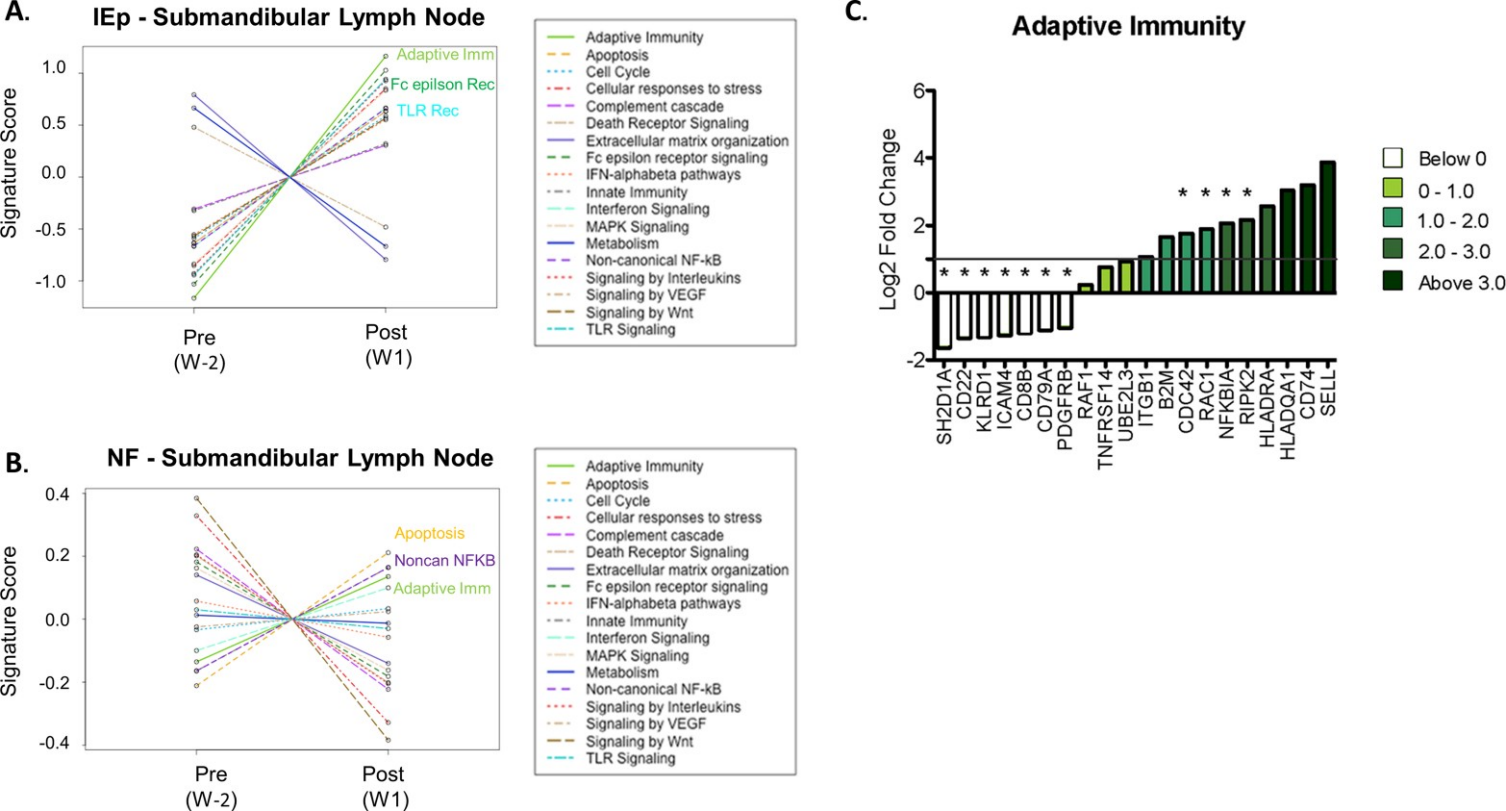
Transcriptomic assessment at lymph nodes and oral mucosa. (A-B) Submandibular lymph nodes extracted pre-immunization at week -2 and post-immunization at week 1 were used to assess the transcriptomic profile by Nanostring transcriptomics. Differential gene expression was determined by comparing week -2 transcript levels to week 1 levels in each macaque. (A-B) Using the advanced analysis package in Nanostring NSolver software, pathway scores were generated by comparing differential genes expression for genes known to function in specific pathways for each macaque within an immunization group, iEp immunization group (A) or NF immunization group (B). The top three pathways showing a positive signature a denoted on the plot. (C) Oral buccal cytobrushes were collected week 1 following the first immunization in both oral iEp vaccinated macaques and in systemic IM vaccinated macaques. Differentially expressed genes were determined by comparing the gene signature in iEp immunization to IM immunization. Log_2_ fold change of genes involved in adaptive immunity in iEp vaccinated macaques. Genes showing statistical significance by are denoted with an asterisk and are denoted on the volcano plot by green boxes.

To investigate the local immune response triggered by the iEp vaccination, the transcriptome was evaluated in buccal mucosa samples collected by oral cytobrush and in the draining submandibular lymph nodes. Concatenating the observed gene signature into a Signature Score, the submandibular lymph node demonstrated changes of gene transcripts associated with adaptive immunity in both the intraepithelial and needle-free macaques (Fig 2A and 2B). In addition, a large number of changes were also observed within the Fc epsilon receptor (iEp), TLR receptor (iEp), apoptosis (NF) and Non-canonical NFkB pathway (NF). These findings indicate that the vaccine administration resulted in global change to the gene transcripts, with a large effect on genes associated with the adaptive immune response for both modes of vaccine delivery.

The global transcriptomic profile in the buccal mucosa of iEp macaques was assessed one week after the first vaccination and compared to that of IM vaccinated macaques to identity differentially expressed genes (volcano plot, S3 Fig). To assign higher level biological meaning to these alterations, pathway analysis was conducted and determined that many of the transcripts were associated with adaptive immunity (Fig 2C). Other potential pathways induced by iEp vaccination include: NFkB (NFkB1A (alpha subunit of IKK protein complex) and RIPK2 (Receptor-Interacting serine/threonine Protein kinase 2) and cell cycle regulation (CDC42 and RAC1). Conversely, iEp immunization significantly down-regulated four gene transcripts that are generally associated with different immune cell subsets: KLRD1 (NK cells), CD8b (T cells) as well as CD22 and CD79A (B cells), which could indicate cell egress from the oral mucosa to other sites for induction of adaptive responses. From these data we conclude that although the oral mucosa is predisposed for immune tolerance due to high antigen exposure, oral immunization can overcome this immune anergy to induce potent local immune responses. Most likely the delivery of TLR adjuvants directly into the tissue significantly contributed to the observed induction of immune-associated gene transcripts as both NFkB signaling and adaptive immunity signatures that were observed in the transcriptome are both downstream of TLR activation, highlighting the importance of adjuvant selection in oral vaccination strategies.

### Systemic maturation/activation of blood dendritic cells following vaccination

**Fig 3:**
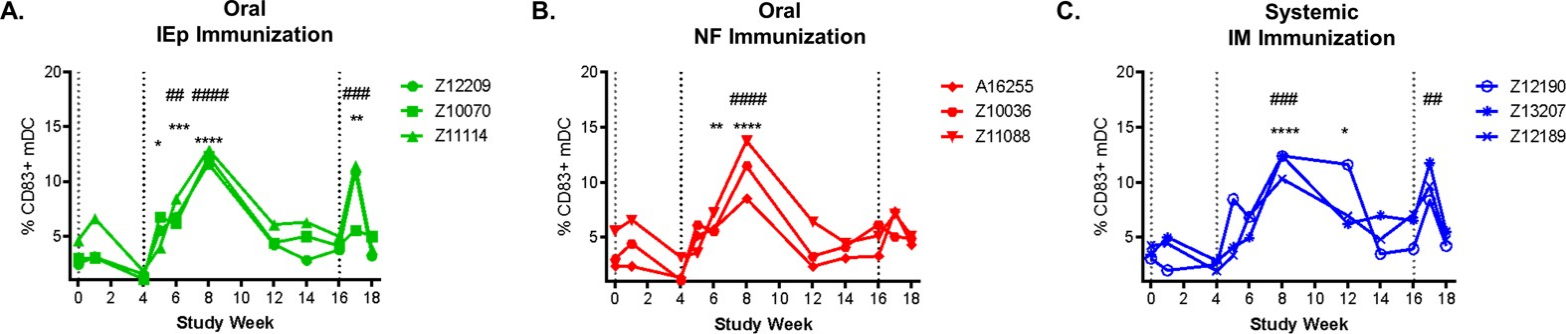
Increase in CD83+ myeloid dendritic cells following vaccination in all immunization groups. The frequency of mature CD83+ myeloid dendritic cells (CD14-CD3-CD20-CD123-HLADR+) was evaluated longitudinally in freshly isolated PBMC for all immunization groups: iEp (A), NF (B), and IM (C). Statistical significance was determined using repeated measures ANOVA with a bonferroni post-hoc test. Timepoints statistically different than week 0 are denoted with #, while timepoints statistically different than the most recent vaccination time point (week 4 or week 16) denoted with *.

The ability of vaccination to influence the activation status of important innate antigen presenting cells (APCs), such as dendritic cells (DCs) and monocytes, within the peripheral blood has been observed following a number of different vaccination strategies [40–42]; however, these responses have been poorly characterized in HIV-1 vaccine studies. Here we focused on how the three different vaccination modalities (iEp, NF and IM) influenced the maturation/activation status of these important APCs in the peripheral blood. The flow cytometry panel and gating strategy is shown in S4 Fig. We observed an increase in CD83+ mature myeloid dendritic cells (mDCs) in circulation following the second vaccine administration for each of the vaccinated macaques in the study regardless of vaccine modality (Fig 3). Overall, these data demonstrate that all three modes of vaccination resulted in comparable changes in the levels of mature mDCs in the peripheral blood, demonstrating that oral vaccination engages the systemic innate immune system similar magnitude and kinetics to systemic IM immunization.

### Similar B cell population kinetics in the peripheral blood following vaccination

**Fig 4:**
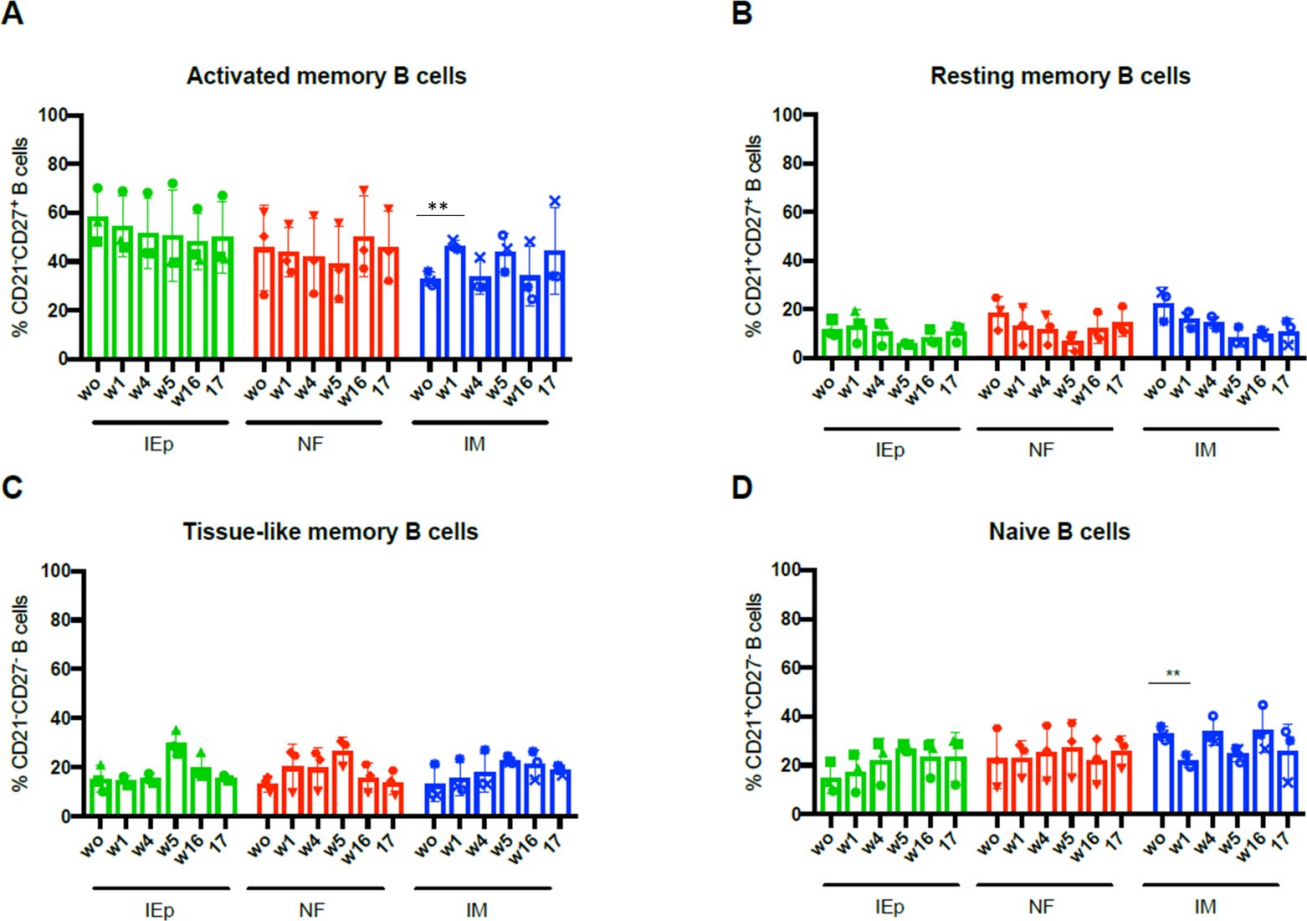
Oral iEP, needle-free and IM immunizations induce similar frequencies of B cell populations. The frequency of activated memory B cells (A), resting memory B cells (B), tissue-like memory B cells (C) and naive B cells (D) was analyzed in the peripheral blood of iEP, needle-free and IM groups at weeks 0,1,4,5,16 and 17. Statistical significance was tested using repeated measures ANOVA with a Bonferroni post-hoc test. ** p≤ 0.01.

Although the innate stimulation in peripheral blood profile was similar in all vaccine groups, we sought to determine whether there might be nuanced differences in how innate immunity engaged the adaptive for the different vaccination strategies. Therefore, we characterized circulating B cell subsets in PBMCs by flow cytometry, staining for the following markers: CD20, CD21, CD27, CD3, IgG, IgM, and IgD. The gating strategy is shown in S5 Fig. We evaluated activated memory B cells (Fig 4A) (CD20^+^, CD21^-^, CD27^+^), resting memory B cells (Fig 4B) (CD20^+^, CD21^+^, CD27^+^), tissue-like memory B cells (Fig 4C) (CD20^+^, CD21^-^, CD27^-^), and naive B cells (Fig 4D) (CD20^+^, CD21^+^, CD27^-^) [43]. These analyses focused on B cell subset frequencies on the day of each immunization (prior to immunization) and one week post each immunization. Within the four B cell subsets, we observed similar levels of circulating B cells for all immunization groups (Fig 4).

Overall, B cell populations in the oral groups remained relatively steady in the samples we analyzed over the course of the immunization trial. As such, it does not appear that oral immunization had a significant impact on peripheral circulating B cell populations. In the IM immunized animals, we detected a statistically significant decrease in naive B cells in the periphery one week after the first immunization (Fig 4D). This decrease was complemented by significantly increased activated memory B cell frequencies in the periphery (Fig 4A). This trend of increased frequency activated memory B cells and decreasing naive B cell frequencies was observed for each immunization when comparing post-immunization samples. These observations imply that IM immunization influenced circulating B cell populations differently than oral immunization, as neither oral method induced measurable changes in peripheral B cell subsets. This contrasts with the observation that similar activation of innate cells in the periphery was observed between groups (Fig 3), indicating that oral and IM immunization may have different systemic effects in peripheral blood.

### Oral vaccination induces robust systemic antibody responses

**Fig 5:**
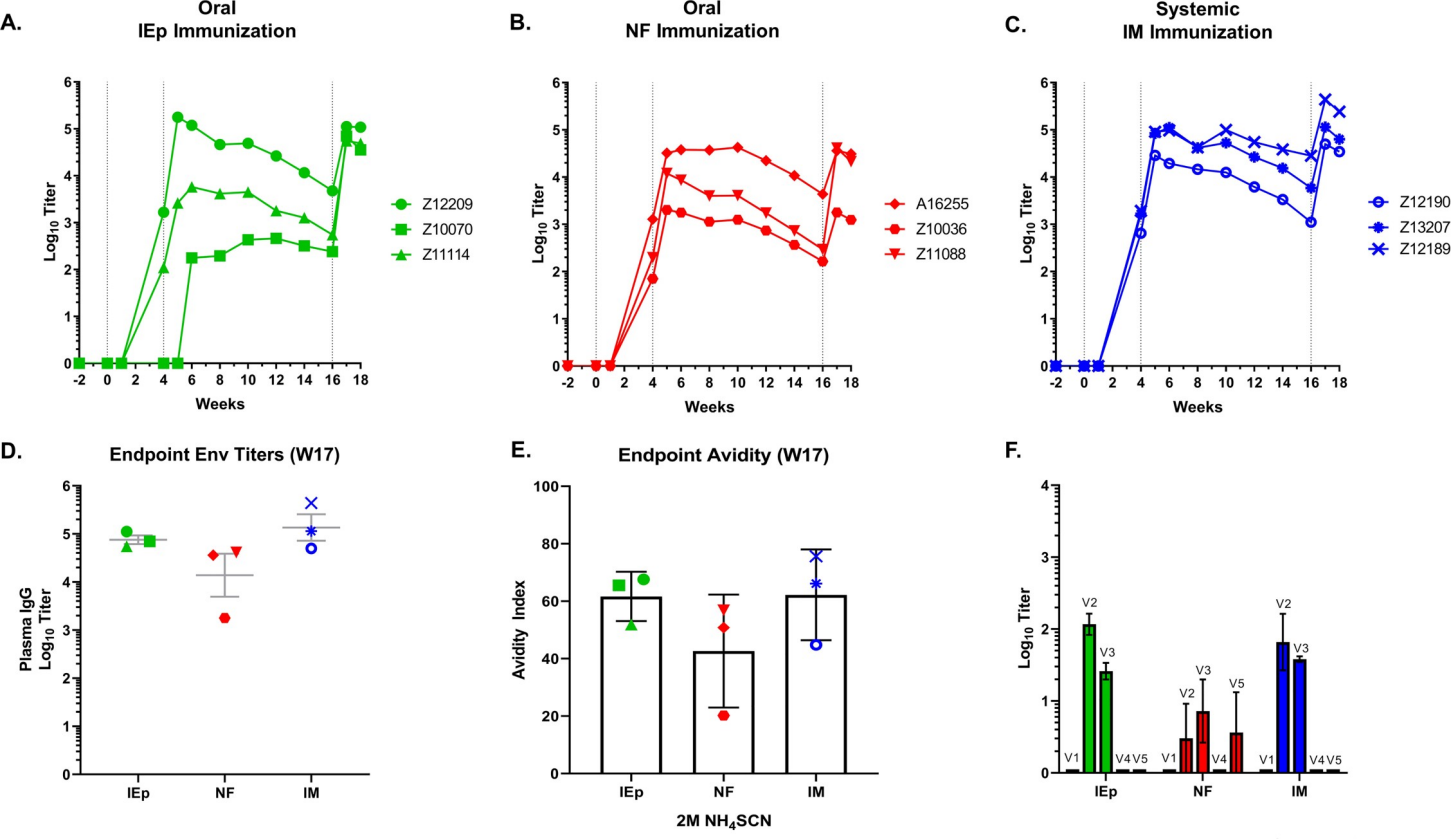
Oral iEp and systemic IM immunization generate comparable Env-specific IgG antibodies in plasma. IgG antibodies to full-length immunogen envelope protein were measured by ELISAs on plasma collected longitudinally for iEp (A), NF (B) and IM (C) vaccinated macaques. Individual macaques are denoted by symbol shape. D) Endpoint week 17 titers for anti-Env plasma IgG antibodies for all immunization groups. E) avidity indices for week 17 plasma against the autologous antigen. F) Binding of plasma antibodies to linear epitopes in the V1, V2, V3, V4, and V5 regions of the 10042.05 envelope sequence.

All three regimens were immunogenic and induced systemic serum IgG antibodies. All routes exhibited similar kinetics and timing in the elicitation and boosting of antibody titers (Fig 5A–5C). Of the treatments, IM showed the least variability in the timing and magnitude of antibody titers over the course of sampling. The oral vaccination routes appeared somewhat more variable, with an endpoint titer spread of 1–3 log_10_ among the animals within a group at some time points. One animal in the iEp group (Z10070) did not appear to respond to the W0 prime but responded to the W4 boost and ended with W17 endpoint titers equivalent to other animals within the group (Fig 5A). It is probable that this disparity was due to variability in the injection procedure, rather than due to the immunization route itself. Overall, endpoint ELISA titers against 10042.05.SOSIP at week 17 were statistically equivalent in all groups, although the NF group trended lower (Fig 5D). We also tested antibody binding avidity to 10042.05.SOSIP, which is an indirect measure of antibody maturation (Fig 5E). The primary binding data is shown in S6 Fig. The endpoint avidity index was statistically similar among groups, although one animal in the NF group (Z10036) had substantially lower avidity to Env. Together, these findings indicate that immunization via the oral mucosal tissues is capable of inducing systemic antibodies with similar binding titers and avidity to vaccination by standard IM routes.

We also evaluated antibody binding against linear peptides corresponding to the variable regions V1-V5 of 10042.05.SOSIP (Fig 5F). Typically, anti-V3 antibody responses are a dominant antibody binding specificity after immunization with Env trimers. Interestingly, the autologous serum anti-V2 antibody responses were greater than, or on par with, anti-V3 antibody responses in all three immunization groups. Binding to the other variable loops (V1, V4, and V5) were negligible, although in the NF group one animal developed anti-V5 antibodies. Together these findings indicate that the V2 is highly immunogenic in 10042.05.SOSIP, and confirm that the modifications to the Env likely dampened responses against the autologous V3 loop.

### Vaccine-elicited anti-Env antibodies mediate phagocytosis

**Fig 6:**
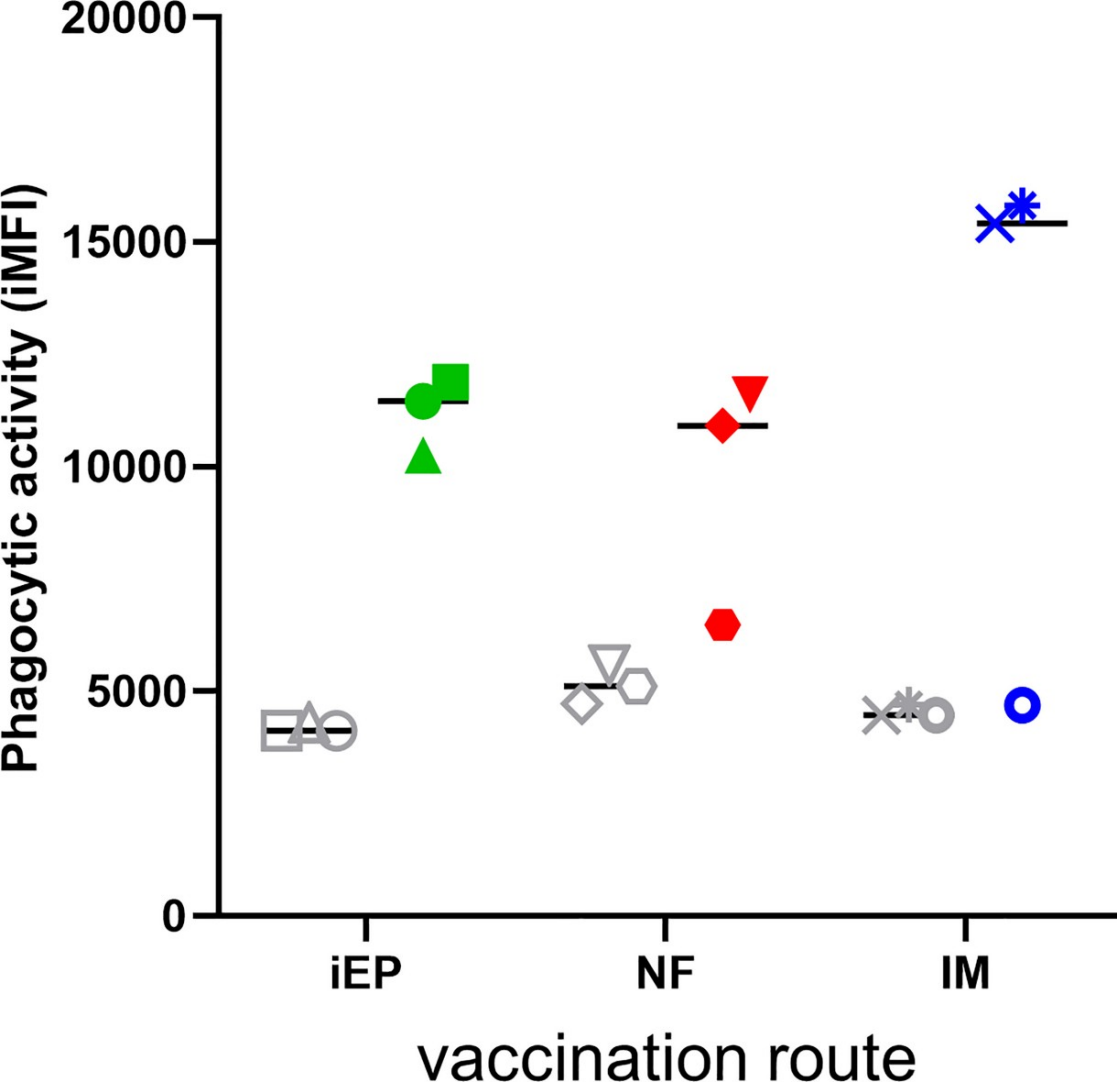
Vaccine-elicited antibodies mediate ADCP. Week 17 (colored symbols) and week 0 pre-bleed (grey symbols) serum samples were assayed for ADCP activity in a bead-based format. The readout is the integrated Mean Fluorescence Intensity (iMFI). Samples are grouped by vaccination route, where iEP = intra-epithelial, NF = needle free, and IM = intramuscular.

Antibody Dependent Cellular Phagocytosis (ADCP) is the process by which antibody-coated foreign objects are actively phagocytosed in a receptor-specific manner through interactions between host Fc receptors and the antibody Fc region [44, 45]. ADCP has been implicated as an important anti-viral process that was associated with protection in the RV144 clinical trial [44]. We evaluated vaccine serum at weeks 0 and 17, the latter being one week post final immunization, in a bead based ADCP assay, as previously described [45]. Animals immunized both orally and IM developed anti-Env antibodies that mediated ADCP, compared to animal-matched pre-bleed controls (Fig 6). Interestingly, immune serum from animals Z10036 (NF group) and Z12190 (IM group) did not exhibit ADCP activity over background. Intriguingly, animal Z10036 had the lowest anti-Env binding titers in the NF group by approximately 1 log_10_ serum dilution^-1^. Animal Z12190 had the lowest binding titers in the IM group, but had similar binding titers to most other animals in all groups (Fig 4D), indicating that the lack of ADCP activity was not due to the magnitude of antibody anti-Env binding titers alone. Overall, 7/9 animals developed ADCP activity in response to vaccination with activity similar in magnitude to previous reports [46].

### Immunization with 10042.05.SOSIP induced cross-reactive anti-V1V2 antibodies

**Fig 7:**
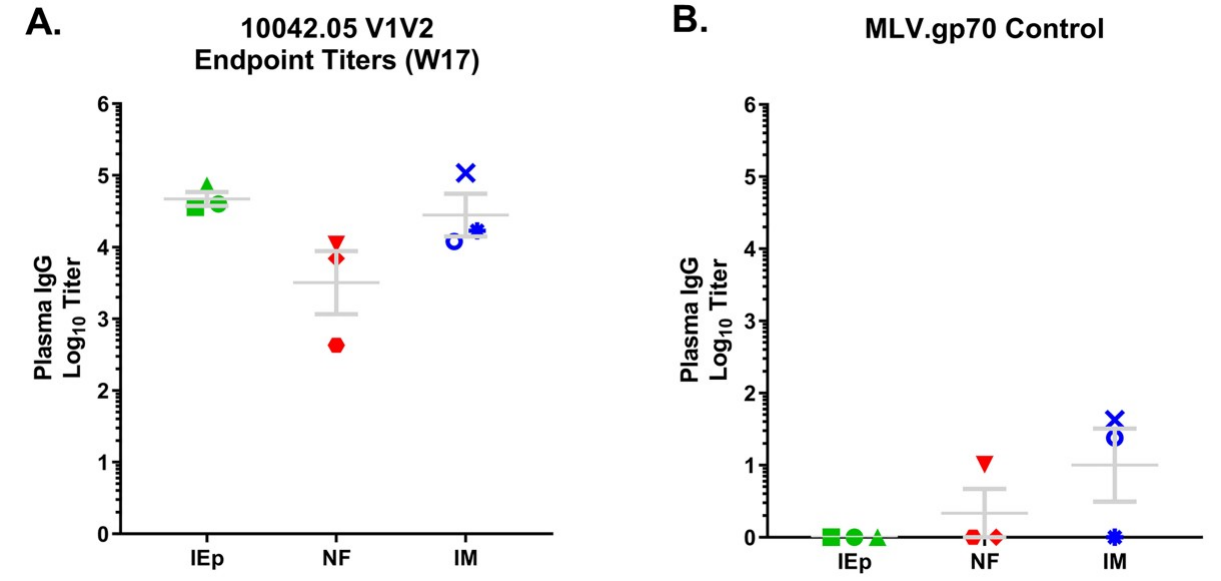
Vaccine-induced bind to the V1V2 region. A-B) Endpoint titers (Log10^-1^) at week 17) for all immunization groups against V1V2-gp70 were measured by ELISAs (A) and compared to background using gp70 alone (B).

When the previous fused gp140 version of 10042.05 was evaluated in rabbits, it elicited high levels cross-clade reactive anti-V1V2 antibodies, and overall the Env loop responses appeared to bias toward the V2 region [31]. To assess whether 10042.05.SOSIP elicited similar responses in NHPs, we evaluated W17 endpoint titers against an MLV-gp70V1V2 construct bearing the autologous V1V2 (MLV-gp70-10042.05V1V2) (Fig 7A). In all assays the gp70 backbone was used as a control for non-specific binding (Fig 7B). We measured high titers of autologous MLV-gp70-10042.05V1V2 antibodies in all groups, although the NF group averaged approximately a log lower response than the other groups. Intriguingly, the anti-V1V2 response comprised a significant portion of the overall polyclonal anti-Env antibody response. Comparatively, the average autologous 10042.05.SOSIP endpoint titer was 1:52,119, whereas the average autologous MLV-gp70-10042.05V1V2 titer among groups was 1:16,126. Thus, on average approximately 31% of antibody binding was attributable to the V1V2 region. This contribution varied, ranging from ~21% in the IM group to ~62% in the iEp group. Regardless of group, these findings indicate that antibodies that recognize the V1V2 region make up a substantial portion of the total circulating anti-Env antibodies elicited by vaccination.

**Fig 8:**
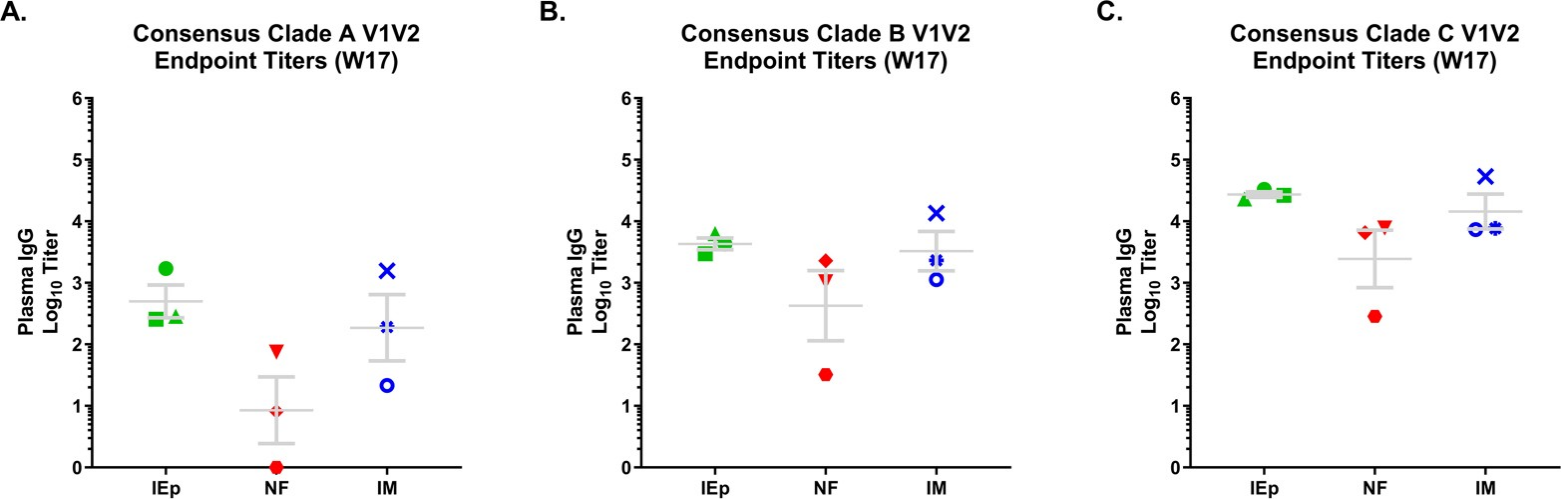
Cross-clade V1V2-specific antibodies. V1V2 consensus sequences for clade A, B, and C (panels A-C, respectively) viruses were constructed and used to determine cross-clade reactivity of V1V2 antibodies for all immunization groups using week 17 plasma by ELISA. Values reported are the Log10^-1^ titers.

We also tested for cross-reactivity by evaluating binding to constructs derived from consensus sequences from Clades A, B, and C. As in our previous study, 10042.05.SOSIP elicited cross reactive anti-V1V2 antibodies against all three clades tested. Titers were lowest against clade A, with average endpoint titers of ~1:100 (Fig 8A), followed by Clade B with an average titer of 1:1820 (Fig 8B). Titers were substantially higher against both clade C V1V2 (1:9840) and the autologous 10042 V1V2 (1:16,126) (Fig 8C and 8A, respectively), both of which were statistically higher than clades A and B V1V2 antibodies.

We evaluated week 17 serum samples for neutralizing activity in the TZM-bl luciferase assay. Our initial analysis focused on more neutralization sensitive Tier 1 isolates from clades A, B, and C, where we screened plasma at a dilution of 1:50 (S7 Fig). In several replicate assays we failed to detect neutralization over 50% against any isolate, even against lab adapted, easy to neutralize viral isolates that are highly sensitive to V3-directed antibodies. This despite the immunogen containing the apical GPGR amino acid motif common to V3-directed neutralizing antibodies. The lack of anti-V3 neutralizing activity is intriguing and implies that the V3 stabilization disulfide bond dampened immune responses and inhibited the generation of anti-V3 neutralizing antibody responses. Further, these results show that the anti-V1V2 responses described above do not have neutralizing activity.

## Discussion

Here we utilized a native-like trimer derived from an elite neutralizer, 10042.05.SOSIP, to evaluate the induction of anti-HIV Env antibody responses following different oral mucosal vaccination of Rhesus macaques. Both the iEp and NF methods were tested and compared to IM injection of the identical vaccine formulation. Oral vaccine delivery resulted in the initiation of innate and adaptive immune response in a manner similar to IM delivery approach, although differences in the peripheral B cell compartment were noted. Importantly, irrespective of oral delivery method 10042.05.SOSIP induced high titers of anti-V1V2 antibodies, indicating an immunogenic bias toward the V1V2 regions. Finally, the iEp oral delivery method was determined to be best at inducing anti-V1-V2 antibodies.

One aspect of this study was to transcriptionally evaluate the earliest/innate immune response that were occurring at two different sites following the oral administration of the vaccine. We reasoned that the one-week post-vaccination timepoint would reveal key changes in the transcriptomic profile of innate immune cells at these two sites. The submandibular lymph nodes revealed an upregulation of a number of key pathways, with the genes associated with the adaptive immune response upregulation in both the iEp and NF vaccinated macaques. Further, evaluation of oral mucosal cheek tissue adjacent to the site of vaccination revealed an upregulation of gene transcripts associated with NFkB and cell cycle regulation likely reflecting an activation of immune cells due to the adjuvants (R848 and MPLA) utilized. Surprisingly, there was a down-regulation of gene transcripts associated with a number of key immune cell subsets (NK cells, B cells, T cells) at this site at 1-week post-infection. This could be attributed to these cells migrating from the mucosa to initiate the adaptive immune response in secondary lymphoid organs, such as the draining lymph node, where indeed a strong adaptive immune signature was observed. These early transcriptomic changes provide insights into the impact of the vaccine/adjuvant and how the vaccination at the oral mucosa sets the stage for the induction of the adaptive immune response, providing confidence that the oral mucosa was a good option for use as a site of vaccine delivery.

Both IM and oral immunization induced mDC maturation in peripheral blood, whereas only IM immunization induced measurable changes in the peripheral blood B cell subsets. Thus, although oral immunization induced systemic innate stimulation, it did not result in changes in the circulating peripheral B cell populations, despite inducing similar IgG titers as IM immunization. Further, oral immunization induced antibodies with similar binding avidity to IM immunization, indicating that both immunization routes induced a maturing antibody response. Thus, both routes are capable of inducing robust class-switched antibody responses, and presumably both immunization routes resulted local germinal center maturation, plasmablast tracking to the bone marrow, and the deposition of long-lived plasma cells to secrete serum IgG. This disparity in peripheral memory B cell proliferation may reflect that the cycles of B cell proliferation and maturation were somewhat compartmentalized in the oral lymphoid tissue, or that B cell trafficking in these tissues fundamentally differs from peripheral lymphoid tissue.

The lack of neutralizing antibodies was not particularly surprising based on previous studies, where thus far SOSIP Envs have not been successful in eliciting broad and potent heterologous neutralizing activity [47–51], and the extreme neutralization resistant phenotype of the parental 10042.05 virus [33]. However, our observation that immunization with 10042.05.SOSIP reliably elicited antibody responses biased toward the V1V2 regions is intriguing, and indicates that this Env may have utility as a vaccine aimed at inducing V1V2 responses. The fact that the fused and SOSIP versions of this Env both induced V1V2 antibodies implies that this Env is intrinsically structurally tuned to bias antibody responses toward the V1V2 region, rather than being a consequence of the assembly of this Env into native-like trimeric structure. Further, the low levels of vaccine-elicited anti-V3 antibodies also indicates that stabilization of the V3 in the SOSIP trimer was successful in dampening anti-V3 responses as measured by peptide binding. Thus far, research on V1V2 antibody responses, such as those induced in RV144, indicate that they are likely to mostly mediate non-neutralizing activities. Thus, it will be of significant interest to dissect the V1V2 responses that can be induced by 10042.05.SOSIP and to assess their Fc-mediated functional capacity.

While the oral mucosa remains a promising vaccine route, methods for immunizing in the oral mucosa need further refinement. Our comparison of oral immunization methods indicated that the iEp oral needle injection delivery route was superior to the NF approach in eliciting high levels of V1-V2 antibodies. This discrepancy in the two delivery routes is possibly due to variability in delivery of the vaccine utilizing the Injex NF device, which is commonly used to deliver liquid jets into the oral gum tissue that has a different structure and consistency than the cheek mucosal tissue [19]. Thus, it may be that needle free approaches need to be fine-tuned to deliver the vaccines to the proper depth and dispersion for maximal uptake in the subepithelial tissue. Although our results indicate that iEp injection was more reliable as a delivery route, needle delivery in the oral mucosa would likely face resistance to implementation due to needle fear. Thus, the optimization of needle free methodology would likely be important in developing vaccines reliant on oral inoculation.

Our findings from this pilot immunization study indicate that oral vaccination of Env trimers is a viable route for inducing systemic humoral immunity against HIV-1, capable of inducing immune responses similar in magnitude to that of intramuscular routes. Importantly, oral immunization induced robust systemic activation of innate immunity as well as driving relevant activation and transcriptional changes in the local lymph nodes, although we did not detect systemic changes in the circulating B cell populations. The lack of proliferation in the peripheral memory B cell compartment after oral mucosal immunization, despite robust antibody responses, will require further investigation, as does why IM was not superior in inducing peripheral antibody responses. Our ongoing studies are focused on evaluation of whether oral immunization results in antigen specific phenotypic differences in B stimulation and maturation, as well as assessing Fc-mediated effector functions of the V1V2 antibodies. Overall, our results indicate that the lymphoid rich oral mucosal tissues may be a powerful entry point into the immune system and warrants further investigations into how the induction of adaptive immunity in the oral mucosa can be harnessed to drive protective antibody responses against HIV-1.

## Materials and methods

### Animal care and ethics statement

All animal studies were conducted in accordance with protocol 4213–04 approved by the Washington National Primate Research Center Institutional Animal Care and Use Committee. All macaques in this study were managed according to the laws, regulations, and guidelines set forth by the United States Department of Agriculture, Institute for Laboratory Animal Research, Public Health Service, National Research Council, Centers for Disease Control, the Weatherall Report titled “The use of nonhuman primates in research”, and the Association for Assessment and Accreditation of Laboratory Animal Care (AAALAC) International. The nutritional plan consisted of standard monkey chow supplemented with a variety of fruits, vegetables, and other edible objects as part of the environmental enrichment program established by the Behavioral Management Unit. In addition, other means of enrichment were delivered and overseen by veterinary staff with animals having access to more than one category of enrichment. Macaques were housed as pairs with any paired macaques exhibiting incompatible behaviors managed by the Behavioral Management staff. Primate health was monitored daily by trained staff. All efforts were made to minimize suffering through the use of minimally invasive procedures, anesthetics, and analgesics when determined appropriate by veterinary staff.

### Envelope immunogen

The VC10042 envelope sequence was identified and cloned from a HIV+ patient from the Vanderbilt cohort, previously described [32, 33]. This sequence was modified as follows for production as soluble gp140 trimeric immunogen. To enhance cleavage, the primary site was changed from REKR to RRRRRR [38, 52] and a stop codon that improves trimer homogeneity and solubility [53] was added at position 683(HXB2 numbering) by site-directed mutagenesis(Agilent Technologies(Santa Clara, CA). VC10042 gp120 sequence was grafted onto a BG505 gp41 tail using a SOSIP motif previously described [35, 47]. In brief, a disulfide bond was introduced between the gp120 and gp41(A501C/T605C) along with the gp41(I559P) and V3(I559P/L201C/A433C) conformational point mutations. Additional gp120-gp41 interface stabilizing mutations 543N, 553S, 588R, 662A and a V3 stabilizing engineered disulfide bond(L201C/A433C) were added [54]. The native leader sequence was replaced with the tissue plasminogen activator (tPA) and the construct was subsequently cloned into a pcDNA3.4(Thermo Fisher, Waltham, MA) expression construct containing a Woodchuck Posttranscriptional Regulatory Element to increase secretion and expression respectively, in mammalian cell culture.

### Expression and purification of 10042.05.SOSIP

The trimeric protein was transiently expressed as a soluble protein in HEK 293F cells transfected with expression plasmids using PEI Max (Polysciences Inc., Warrington, PA), as previously described [31]. The cultures were harvested six days post transfection and the supernatant was clarified by centrifμgation. The soluble proteins were purified by affinity chromatography using agarose bound *Glanathus nivalus* lectin (Vector Laboratories, Inc., Burlingame, CA) and eluted using 20mM TRIS pH 7.4, 100mM NaCl, 1mM EDTA, containing 1M Methyl α-D-mannopyranoside. The lectin purified protein was buffer exchanged into 20mM TRIS 100mM NaCl, pH 8.0 and ion exchange chromatography (IEX) was performed using a HiTRAP Capto DEAE column to remove aggregate species. The IEX purified fractions were subsequently concentrated and trimer species were resolved by SEC using a Superdex 200 16/600 pg column. SEC fractions were run by Native-PAGE to visualize trimeric species prior to final immunogen pooling.

### Octet BioLayer interferometry

Binding experiments were carried out using an Octet® QKe System (FortéBio, Fremont, CA). Ligands and analytes were diluted in buffer comprised of PBS pH 7.4 containing 0.02% Tween-20, 0.1mg/mL BSA, and 0.05% sodium azide. Monoclonal antibodies at a concentration of 10μg/mL were immobilized on anti-human IgG Fc sensors for 20 seconds. These sensors were then immersed into SOSIP trimer at a concentration of 100μg/mL for a 200 seconds association phase followed by a 200 seconds dissociation phase in buffer alone. All steps were carried out with shaking at 1000 rpm.

### Animal immunizations

All animal studies were conducted at the Washington National Primate Research Center under under protocols approved and monitored by the University of Washington IACUC. Three groups of n = 3 *Macaca mulatta* were immunized at weeks 0, 4, and 16 with 100μg of 10042.SOSIP. The immunogen was adjuvanted with 100μg Resiquimod (R848) (InvivoGen, San Diego, CA), 10μg synthetic monophosphoryl lipid A (MPLA) (PHAD®) (Avanti Polar Lipids Inc., Alabaster, Alabama), and 0.1% Alhydrogel (InvivoGen, San Diego, CA). Each group received the same amount of immunogen and the same vol/vol percentage of adjuvant, however the volume administered varied depending on route of immunization. The intramuscular (IM) immunized group received a 250μL injection into each tricep for a total volume of 500μL containing the above formulation. Animals receiving intraepithelial (IE) or Needle Free (NF) injections were tattooed at a single point in the buccal mucosa in order to deliver the vaccine to the same site at each immunization timepoint. IM animals were similarly tattooed to control for any effects due to this procedure. IE and NF groups received 50μL containing the above formulation at the tattoo site by needle injection or by the Injex 30^™^ needle free injection system (Injex^™^ Equidyne Systems, UK), respectively. IE injections were carried out by inserting a 26-gauge needle at a shallow angle (<45 degrees) into the sub-epithelial tissue and expressing the syringe. NF injections were carried out by placing the loaded Injex mechanism with a spacer at a 90 degree angle and actuating the liquid jet [39, 55].

### ELISA

Plasma IgG titers to autologous 10042.05 envelope were determined using ELISA. Plasma was heat-inactivated for 1 hour at 56°C prior to the assay. Immulon 2HB 96-well plates (Thermo Scientific) were coated with fifty nanograms per well of 10042.05.RM.QSS.V3.SOSIP trimer in 0.1M NaHCO_3_, pH 9.5. Plates were washed between each ELISA step with PBS containing 0.2% Tween-20. Coated plates were blocked with PBS, 10% non-fat milk, and 0.3% Tween-20 for 1 hour at 37°C. Following blocking, plasma samples were serially diluted five-fold over a range of 1:50 to 1:3,906,250 in 100 ul of PBS, 10% non-fat milk, 0.03% Tween-20 and incubated for 1 hour at 37°C. Bound antibodies were detected using goat anti-human IgG (H+L)-HRP (Invitrogen, A18805) diluted 1:1000 in PBS, 10% non-fat milk, 0.03% Tween-20 and incubated for 1 hr at 37°C. Plates were developed using 50 ul of TMB Peroxidase Substrate (SeraCare Life Sciences Inc), then stopped after 3 minutes with an equal volume of 1N H_2_SO_4_. Absorbance at 450 nm was determined with the BioTek ELx800 microplate reader. Endpoint titers were defined as the reciprocal of plasma dilution at O.D. 0.1 after the subtraction of any non-specific signal from pre-immunization plasma.

To measure plasma IgG binding to the HIV V1V2 variable loops, Murine Leukemia Virus (MLV) gp70-V1V2 scaffolds were constructed and purified as previously described [1]. In addition to the consensus clade A, B, C V1V2 constructs, as well as the MLV.gp70 construct alone, we made a variant containing the autologous 10042.05 V1V2 sequence. Animal plasma was serially diluted over a range of 1:10 to 1:163,840 in 60 ul volume and antibody binding to MLV.gp70-V1V2 proteins was measured using ELISA as described above.

To measure plasma IgG binding to the linear epitopes of HIV variable loops, peptides corresponding to the crown regions of V1-V5 loops of 10042.05 envelope were synthesized (GenScript, >85% purity) and coated onto ELISA plates at one hundred nanograms per well. Plasma was serially diluted over a range of 1:10 to 1:21,870 in 100 ul volume and antibody binding to each peptide was measured using ELISA as described above.

### Chaotrope avidity assay

Antibody avidity of post-3rd immunization plasma was measured using the ELISA format described above, with one additional step. Plasma was diluted over a range of 1:50 to 1:3,906,250 and plated in quadruplicate side by side on the same plate. After a 1 hr incubation at 37°C, plates were washed, and half of the sample wells for each sample were treated with 2M NH4SCN in PBS, while the other half was treated with PBS alone. Plates were incubated for 15 mins at room temperature and then washed. Bound antibodies were detected using the goat anti-human IgG secondary antibody as above. The avidity index was calculated as the ratio of AUC of samples treated with the chaotrope over AUC of samples without the chaotrope: (AUCNH4SCN / AUCPBS) × 100.

### Antibody dependent cellular phagocytosis

Antibody Dependent Cellular Phagocytosis (ADCP) was assessed as previously described [45], with minor modifications. Briefly, 1 μm fluorescent NeutrAvidin beads (Invitrogen, F8776) were coated with biotinylated 10042.05 gp120 overnight at 4°C. Plasma was heat-inactivated for 1 hour at 56°C and incubated at 1:50 dilution with 2 x 10^6^ beads for 2 hours at 37°C in a 96-well U-bottom plate (Corning, 353227). THP-1 monocytes (ATCC^Ⓡ^ TIB-202^TM^) were cultured as recommended by ATCC and 20,000 cells were added to each well in 100 μl volume. The plates were incubated overnight at 37°C to allow for phagocytosis. The following morning cells were fixed with 100 μl of 4% PFA (Electron Microscopy Sciences, 15710) and analyzed on the BD LSRII flow cytometer. Assay readout is represented by the integrated Mean Fluorescence Intensity (iMFI), which is the product of the geometric mean fluorescence intensity and the frequency of bead-positive (FITC+) cells.

### Neutralization assays

Neutralizing antibody activity against a Tier 1 panel of pseudoviruses from clades A, B, C was measured using the TZM-bl cell-based neutralization assay as previously described [2, 31], with minor modifications. Briefly, TZM-bl cells were plated at a density of 4 x 10^3^ per well of a 96-well plate (Falcon, 353075) 24 hours prior to the assay, and polybrene reagent was added to the cells at 2 ug/ml 30 minutes prior to inoculation with test samples. Heat-inactivated plasma at 1:50 final dilution was pre-incubated with pseudovirus for 90 minutes at 37°C. Following the incubation, polybrene-containing medium was aspirated and the virus/plasma mixture was added to the TZM-bl cells. After 72 hours at 37°C, cell supernatant was replaced with 100 ul of Promega Steady Glo reagent, cells were allowed to lyse for 5 minutes at room temperature, and luciferase activity was measured as relative light units (RLU) using a Fluoroskan Ascent FL luminometer. RLU values used to determine percent neutralization are the average of triplicate wells of pre and post immune plasma samples tested on the same plate. Percent neutralization was calculated using the following equation: (RLU_pre_ − RLU_post_) ÷ (RLU_pre_ − RLU_cell background_) × 100. To test for nonspecific neutralizing activity, we used MLV envelope pseudovirus and subtracted three times any noted MLV neutralization values from all other neutralization results for a given plasma sample.

### Plasma and PBMC isolation

For plasma isolation, whole blood collected in EDTA anti-coagulant tubes was centrifuged at 1400 rpm for 10 minutes in a Sorvall Legend Centrifuge. Plasma was aliquoted and stored at -80°C until use. Peripheral blood mononuclear cells (PMBCs) were then obtained by diluting the remaining blood in 1:1 in D-PBS and layering over 90% Ficoll/10% D-PBS. Ficoll gradients were centrifuged in a Sorvall Legend Centrifuge for 20 minutes at 2500 rpm without a brake. The PBMC layer was removed and placed in a fresh 50 mL conical tube and washed with 50 mL D-PBS twice. After the final wash, PBMCs were resuspended in 10 mL D-PBS and enumerated with a hemocytometer. One million cells were removed for flow analysis and the remaining PBMCs were stored in 90% FBS/10% DMSO at ten million cells/mL in liquid nitrogen.

### Submandibular lymph node processing

Submandibular lymph nodes were excised two weeks prior (W-2) and one week (W1) after the first immunization and immediately placed in 5 mL RPMI on ice. For processing, lymph nodes were placed in a 70um strainer inside a 50 mL conical tube and smashed to release the cells. After washing the strainer to remove any residual cells, the collected cells were pelleted by centrifugation for 7 minutes at 1400 rpm and enumerated. One million cells were immediately placed in 350 uL RA1 buffer containing 1% beta-mercaptoethanol for transcriptomic analysis. The remaining cells were preserved in 90% FBS/10% DMSO at 10 million cells/mL in liquid nitrogen.

### Oral cytobrush processing

Oral biopsy cytobrushes (Oral Cdx Laboratories) were used to collect samples from the cheek one week following vaccination by turning the cytobrush ten times along the buccal mucosa and immediately placing the brush in 1 mL RA1 buffer containing 1% beta-mercaptoethanol for transcriptomic profiling. Samples were stored at -80°C for RNA extraction.

### Flow cytometry analysis of PBMC

For flow cytometry analysis, one million total PBMCs were stained for 30 minutes in the dark with a mixture of antibodies. The staining panel consisted of Live/dead Violet Fixable Dead Stain (diluted 1:500 in PBS, Life Technologies), CD3-Pacific blue (clone SP34-2, BD Biosciences), CD20-BV570 (clone 2H7, Biolegend), CD16-BV605 (clone 3G8, Biolegend), CD86-BV711(clone 2338, BD Biosciences), HLADR-PE (clone G46-6, BD Biosciences), CD83-PE-CF594 (clone HB15e, BD Biosciences), CD123-PerCP-Cy5.5 (clone 7G3, BD Biosciences), CD80-PECy7 (clone L307.4, BD Biosciences), CD11c-APC (clone S-HCL-3, BD Biosciences), CD14-APC-H7 (clone m5E2, BD Biosciences), and Brilliant Stain Buffer (BD Biosciences). Following staining, each sample was washed once with D-PBS containing 2% FBS, pelleted by centrifugation and then resuspended in 250 uL of 1% PFA. Cells were acquired on a BD LSRII flow cytometer. Data were analyzed using FlowJo software (version 1.1.0-SNAPSHOT). Gates for cell populations were determined using fluorescence minus one (FMO) stained controls.

For B cell analyses, cryopreserved PBMCs were thawed, counted and one million cells were stained for the following: Live/dead Violet Fixable Dead Stain (diluted 1:500 in PBS, Life Technologies), CD3-Pacific Blue (clone SP34-2, BD Biosciences), CD20-BV570 (clone 2H7, BioLegend), CD21-BV711 (clone B-ly4, BD Biosciences), CD27-BV785 (clone O323, BioLegend), IgM-BV605 (clone G20-127, BD Biosciences), IgD-Alexa 488 (clone IA6-2, BioLegend) IgG-APC H7 (G18-145, BD Biosciences), and Brilliant Stain Buffer (BD Biosciences) for 30 minutes in the dark. Following staining, samples were washed with PBS containing 2% FBS, centrifuged for 7 min at 1200 rpm and then resuspended in 250 uL 1% PFA. Data was acquired on a BD LSRII flow cytometer and analyzed using FlowJo software. The gating strategy was determined using FMO controls.

### Nanostring transcriptomic analysis

Oral cytobrush samples and cells isolated from submandibular lymph nodes were used for RNA extraction using a NucleoSpin RNA isolation kit (Macherey-Nagel). RNA was diluted to 20 ng/uL in nuclease-free water and used for transcriptomic analysis using a Nanostring Non-human Primate Immunology Consortium Panel (Nanostring, Seattle, WA). Probe set-target RNA hybridization reactions were performed according to the manufacturer’s protocol using 100 ng (5 uL) of total RNA. Purified probe set-targets were processed and immobilized on nCounter Cartridges using an nCounter MAX prep station. Transcripts of interest were quantified on the Digital Analyzer for each sample. For data analysis, nCounter.RCC files were imported in nSolver Analysis Software 4.0 and checked for quality control. Determination of differentially expressed genes, pathways analysis and cell profiling were conducted using the Nanostring Advanced Analysis software per the manufacturer’s instructions. For the oral cytobrush samples, differentially expressed genes were determined by comparing the normalized count data between iEp immunized macaques to IM immunized macaques. The volcano plot depicting Log2 fold changes and genes with unadjusted p values < 0.05 was constructed using R studio (v 1.1.463). For submandibular lymph node samples, differentially expressed genes were determined by comparing transcript levels at week 1 vs week -2 for all three immunization groups.

### Statistical analysis

Statistical analyses were performed using Prism version 5.0f (GraphPad software, Inc). The specific tests used to analyze each data set are indicated in the figure legends. For longitudinal cell frequencies, significance was determined using a one-way ANOVA followed by the Bonferroni’s post-hoc multiple comparisons test. In all cases, p values < 0.05 were considered significant.

## Supporting information

S1 Fig SOSIP Env QC and binding to trimer-specific mAbs. A) Size exclusion chromatography (SEC) profile of 10042.05.SOSIP following lectin affinity purification and ion exchange chromatography. B) Blue native PAGE of final trimer preparation.
(TIF)

S2 Fig Binding profile of 10042.05.SOSIP to trimer-specific mAbs PGT151and 8ANC195 (A), CD4-IgG2 (B), bNAbs PG16 and VRC01, and the V3-directed mAb 447-52D, as determined by biolayer interferometry(BLI). In (A), human EPCR protein is shown in a red trace as a negative control.
(TIF)

S3 Fig Volcano plot. Oral buccal cytobrushes were collected week 1 following the first immunization in both oral iEp vaccinated macaques and in systemic IM vaccinated macaques. Differentially expressed genes were determined by comparing the gene signature in iEp immunization to IM immunization. Global transcriptome volcano plot showing genes with a 0.5 log_2_ fold change and p ≤ 0.05 in iEp immunized macaques compared IM immunized macaques denoted in red. Statistical significance was determined by t-tests.
(TIF)

S4 Fig Innate cell flow cytometry gating strategy. A) Stained PBMCs were first gated on single, live, CD3- cells, followed by identification of myeloid dendritic cells (mDCs) using the markers HLADR+CD14-CD20-CD11c+. B) mDCs were then phenotyped using the markers CD80, CD86 and CD83.
(TIF)

S5 Fig The gating strategy used to define the B cell populations in the peripheral blood consisted of gating on singlets **(A)**, then lymphocytes **(B)**, followed by exclusion of dead/CD3^+^ cells **(C)**. The surface markers CD21 and CD27 were used to distinguish the following B cell subsets: activated memory (CD20^+^CD21^-^CD27^+^), resting memory (CD20^+^CD21^+^CD27^+^), tissue-like memory (CD20^+^CD21^-^CD27^-^) and naive (CD20^+^CD21^+^CD27^-^) **(D)**. The expression of surface immunoglobulin M (IgM) and D (IgD) within each B cell subset was determined as shown **(E)**. The expression of surface immunoglobulin G (IgG) was determined by first gating on the IgD^-^IgM^-^ population, followed by gating on the IgG^+^ population **(F)**.
(TIF)

S6 Fig Chaotrope avidity of envelope-specific plasma IgG—binding curves. Avidity of envelope-specific IgG (week 17) was measured by ELISA using 2M ammonium thiocyanate (NH_4_SCN) treatment. Individual macaques are denoted by symbol color and shape, NH_4_SCN-treated samples are indicated by dashed lines, PBS-treated samples are indicated by solid lines.
(TIF)

S7 Fig Neutralization Panel of Tier 1 isolates. Plasma from week 17 (1 week post 3rd immunization) were tested for neutralizing activity in the TZM-bl assay. Plasma were tested a dilution of 1:50 in triplicate wells and compared against virus-alone entry. Each data point represents the average of triplicate wells. The viruses derive from clades A, B, and C, and are known to have a tier 1, easy to neutralize phenotype. The standard cutoff of 50% is noted by a dotted line.
(TIF)

## References

[1] Rerks-Ngarm S, Pitisuttithum P, Nitayaphan S, Kaewkungwal J, Chiu J, Paris R, et al Vaccination with ALVAC and AIDSVAX to prevent HIV-1 infection in Thailand. N Engl J Med. 2009;361(23):2209–20. 10.1056/NEJMoa0908492 19843557

[2] Haynes BF, Gilbert PB, McElrath MJ, Zolla-Pazner S, Tomaras GD, Alam SM, et al Immune-correlates analysis of an HIV-1 vaccine efficacy trial. N Engl J Med. 2012;366:1275–86. 10.1056/NEJMoa1113425 PubMed Central PMCID: PMC3371689. 22475592 PMC3371689

[3] Yates NL, Liao H-X, Fong Y, deCamp A, Vandergrift NA, Williams WT, et al Vaccine-induced Env V1-V2 IgG3 correlates with lower HIV-1 infection risk and declines soon after vaccination. Sci Transl Med. 2014;6(228):228ra39. 10.1126/scitranslmed.3007730 PubMed Central PMCID: PMC4116665. 24648342 PMC4116665

[4] Bonsignori M, Pollara J, Moody MA, Alpert MD, Chen X, Hwang K-K, et al Antibody-dependent cellular cytotoxicity-mediating antibodies from an HIV-1 vaccine efficacy trial target multiple epitopes and preferentially use the VH1 gene family. J Virol. 2012;86(21):11521–32. 10.1128/JVI.01023-12 PubMed Central PMCID: PMC3486290. 22896626 PMC3486290

[5] Liu P, Yates NL, Shen X, Bonsignori M, Moody MA, Liao H-X, et al Infectious virion capture by HIV-1 gp120-specific IgG from RV144 vaccinees. J Virol. 2013;87(14):7828–36. 10.1128/JVI.02737-12 PubMed Central PMCID: PMC3700223. 23658446 PMC3700223

[6] Zolla-Pazner S, deCamp AC, Cardozo T, Karasavvas N, Gottardo R, Williams C, et al Analysis of V2 antibody responses induced in vaccinees in the ALVAC/AIDSVAX HIV-1 vaccine efficacy trial. PLoS One. 2013;8(1):e53629 10.1371/journal.pone.0053629 PubMed Central PMCID: PMC3547933. 23349725 PMC3547933

[7] Tomaras GD, Ferrari G, Shen X, Alam SM, Liao H-X, Pollara J, et al Vaccine-induced plasma IgA specific for the C1 region of the HIV-1 envelope blocks binding and effector function of IgG. Proc Natl Acad Sci U S A. 2013;110(22):9019–24. 10.1073/pnas.1301456110 PubMed Central PMCID: PMC3670311. 23661056 PMC3670311

[8] Liao H-X, Bonsignori M, Alam SM, McLellan JS, Tomaras GD, Moody MA, et al Vaccine induction of antibodies against a structurally heterogeneous site of immune pressure within HIV-1 envelope protein variable regions 1 and 2. Immunity. 2013;38(1):176–86. 10.1016/j.immuni.2012.11.011 PubMed Central PMCID: PMC3569735. 23313589 PMC3569735

[9] Tay MZ, Liu P, Williams LD, McRaven MD, Sawant S, Gurley TC, et al Antibody-Mediated Internalization of Infectious HIV-1 Virions Differs among Antibody Isotypes and Subclasses. PLoS Pathog. 2016;12(8):e1005817 10.1371/journal.ppat.1005817 PubMed Central PMCID: PMC5007037. 27579713 PMC5007037

[10] Hessell AJ, Hangartner L, Hunter M, Havenith CE, Beurskens FJ, Bakker JM, et al Fc receptor but not complement binding is important in antibody protection against HIV. Nature. 2007;449:101–4. 17805298 10.1038/nature06106

[11] Hessell AJ, Rakasz EG, Poignard P, Hangartner L, Landucci G, Forthal DN, et al Broadly neutralizing human anti-HIV antibody 2G12 is effective in protection against mucosal SHIV challenge even at low serum neutralizing titers. PLoS Pathog. 2009;5:e1000433 10.1371/journal.ppat.1000433 Epub 2009 May 15.; PubMed Central PMCID: PMC2674935. 19436712 PMC2674935

[12] Hessell AJ, Rakasz EG, Tehrani DM, Huber M, Weisgrau KL, Landucci G, et al Broadly Neutralizing Monoclonal Antibodies 2F5 and 4E10, Directed Against the Human Immunodeficiency Virus Type 1 (HIV-1) gp41 Membrane Proximal External Region (MPER), Protect Against SHIVBa-L Mucosal Challenge. J Virol. 2009. 10.1128/JVI.01272-09 PMC2812338 19906907

[13] Mascola JR, Stiegler G, VanCott TC, Katinger H, Carpenter CB, Hanson CE, et al Protection of macaques against vaginal transmission of a pathogenic HIV-1/SIV chimeric virus by passive infusion of neutralizing antibodies. Nat Med. 2000;6:207–10. 10655111 10.1038/72318

[14] McGuire AT, Hoot S, Dreyer AM, Lippy A, Stuart A, Cohen KW, et al Engineering HIV envelope protein to activate germline B cell receptors of broadly neutralizing anti-CD4 binding site antibodies. J Exp Med. 2013;210:655–63. 10.1084/jem.20122824 jem.20122824 [pii] Epub 2013 Mar 25.; PubMed Central PMCID: PMC3620356. 23530120 PMC3620356

[15] Jardine J, Julien JP, Menis S, Ota T, Kalyuzhniy O, McGuire A, et al Rational HIV Immunogen Design to Target Specific Germline B Cell Receptors. Science. 2013. 10.1126/science.1234150 PMC3689846 23539181

[16] McCoy LE. The expanding array of HIV broadly neutralizing antibodies. Retrovirology. 2018;15(1):70 10.1186/s12977-018-0453-y PubMed Central PMCID: PMC6192334. 30326938 PMC6192334

[17] Subbaraman H, Schanz M, Trkola A. Broadly neutralizing antibodies: What is needed to move from a rare event in HIV-1 infection to vaccine efficacy? Retrovirology. 2018;15(1):52 10.1186/s12977-018-0433-2 PubMed Central PMCID: PMC6064177. 30055627 PMC6064177

[18] Kwong PD, Mascola JR. HIV-1 Vaccines Based on Antibody Identification, B Cell Ontogeny, and Epitope Structure. Immunity. 2018;48(5):855–71. 10.1016/j.immuni.2018.04.029 29768174

[19] Shakya AK, Chowdhury MYE, Tao W, Gill HS. Mucosal vaccine delivery: Current state and a pediatric perspective. J Control Release. 2016;240:394–413. 10.1016/j.jconrel.2016.02.014 PubMed Central PMCID: PMC5381653. 26860287 PMC5381653

[20] Creighton RL, Woodrow KA. Microneedle-Mediated Vaccine Delivery to the Oral Mucosa. Adv Healthc Mater. 2019;8(4):e1801180 10.1002/adhm.201801180 PubMed Central PMCID: PMC6476557. 30537400 PMC6476557

[21] Kang SH, Hong SJ, Lee Y-K, Cho S. Oral Vaccine Delivery for Intestinal Immunity-Biological Basis, Barriers, Delivery System, and M Cell Targeting. Polymers. 2018;10(9). 10.3390/polym10090948 PubMed Central PMCID: PMC6403562. 30960873 PMC6403562

[22] Hervouet C, Luci C, Cuburu N, Cremel M, Bekri S, Vimeux L, et al Sublingual immunization with an HIV subunit vaccine induces antibodies and cytotoxic T cells in the mouse female genital tract. Vaccine. 2010;28(34):5582–90. 10.1016/j.vaccine.2010.06.033 20600505

[23] Kraan H, Vrieling H, Czerkinsky C, Jiskoot W, Kersten G, Amorij J-P. Buccal and sublingual vaccine delivery. J Control Release. 2014;190:580–92. 10.1016/j.jconrel.2014.05.060 24911355 PMC7114675

[24] Jones AT, Shen X, Walter KL, LaBranche CC, Wyatt LS, Tomaras GD, et al HIV-1 vaccination by needle-free oral injection induces strong mucosal immunity and protects against SHIV challenge. Nat Commun. 2019;10(1):798 10.1038/s41467-019-08739-4 PubMed Central PMCID: PMC6379385. 30778066 PMC6379385

[25] Matchett WE, Anguiano-Zarate SS, Nehete PN, Shelton K, Nehete BP, Yang G, et al Divergent HIV-1 Directed Immune Responses Generated by Systemic and Mucosal Immunization with Replicating Single-Cycle Adenoviruses in Rhesus Macaques. J Virol. 2019 10.1128/JVI.02016-18 30842321 PMC6498041

[26] Mercier GT, Nehete PN, Passeri MF, Nehete BN, Weaver EA, Templeton NS, et al Oral immunization of rhesus macaques with adenoviral HIV vaccines using enteric-coated capsules. Vaccine. 2007;25:8687–701. 18063450 10.1016/j.vaccine.2007.10.030 PMC2225545

[27] McNeilly CL, Crichton ML, Primiero CA, Frazer IH, Roberts MS, Kendall MA. Microprojection arrays to immunise at mucosal surfaces. J Control Release. 2014;196:252–60. 10.1016/j.jconrel.2014.09.028 S0168-3659(14)00671-3 [pii] Epub 2014 Oct 5. 25285611

[28] Brandtzaeg P. Immune functions of nasopharyngeal lymphoid tissue. Adv Otorhinolaryngol. 2011;72:20–4. 10.1159/000324588 21865681

[29] Illum L. Nasal drug delivery—possibilities, problems and solutions. J Control Release. 2003;87(1–3):187–98. 10.1016/s0168-3659(02)00363-2 12618035

[30] Perry M, Whyte A. Immunology of the tonsils. Immunol Today. 1998;19(9):414–21. 9745205 10.1016/s0167-5699(98)01307-3

[31] Carbonetti S, Oliver BG, Glenn J, Stamatatos L, Sather DN. Soluble HIV-1 envelope immunogens derived from an elite neutralizer elicit cross-reactive V1V2 antibodies and low potency neutralizing antibodies. PLoS One. 2014;9:e86905 10.1371/journal.pone.0086905 PONE-D-13-31886 [pii] eCollection 2014.; PubMed Central PMCID: PMC3900663. 24466285 PMC3900663

[32] Sather DN, Armann J, Ching LK, Mavrantoni A, Sellhorn G, Caldwell Z, et al Factors associated with the development of cross-reactive neutralizing antibodies during human immunodeficiency virus type 1 infection. J Virol. 2009;83:757–69. 10.1128/JVI.02036-08 JVI.02036-08 [pii] Epub 2008 Nov 5.; PubMed Central PMCID: PMC2612355. 18987148 PMC2612355

[33] Sather DN, Carbonetti S, Kehayia J, Kraft Z, Mikell I, Scheid JF, et al Broadly neutralizing antibodies developed by an HIV-positive elite neutralizer exact a replication fitness cost on the contemporaneous virus. J Virol. 2012;86:12676–85. 10.1128/JVI.01893-12 JVI.01893-12 [pii] Epub 2012 Sep 12.; PubMed Central PMCID: PMC3497623. 22973035 PMC3497623

[34] Kwon YD, Pancera M, Acharya P, Georgiev IS, Crooks ET, Gorman J, et al Crystal structure, conformational fixation and entry-related interactions of mature ligand-free HIV-1 Env. Nat Struct Mol Biol. 2015;22(7):522–31. 10.1038/nsmb.3051 PubMed Central PMCID: PMC4706170. 26098315 PMC4706170

[35] Sanders RW, Derking R, Cupo A, Julien JP, Yasmeen A, de Val N, et al A next-generation cleaved, soluble HIV-1 Env Trimer, BG505 SOSIP.664 gp140, expresses multiple epitopes for broadly neutralizing but not non-neutralizing antibodies. PLoS Pathog. 2013;9:e1003618 10.1371/journal.ppat.1003618 PPATHOGENS-D-13-01512 [pii] Epub 2013 Sep 19.; PubMed Central PMCID: PMC3777863. 24068931 PMC3777863

[36] Torrents de la Peña A, Julien J-P, de Taeye SW, Garces F, Guttman M, Ozorowski G, et al Improving the Immunogenicity of Native-like HIV-1 Envelope Trimers by Hyperstabilization. Cell Rep. 2017;20(8):1805–17. 10.1016/j.celrep.2017.07.077 PubMed Central PMCID: PMC5590011. 28834745 PMC5590011

[37] Blattner C, Lee JH, Sliepen K, Derking R, Falkowska E, de la Peña AT, et al Structural delineation of a quaternary, cleavage-dependent epitope at the gp41-gp120 interface on intact HIV-1 Env trimers. Immunity. 2014;40(5):669–80. 10.1016/j.immuni.2014.04.008 PubMed Central PMCID: PMC4057017. 24768348 PMC4057017

[38] Sanders RW, Vesanen M, Schuelke N, Master A, Schiffner L, Kalyanaraman R, et al Stabilization of the soluble, cleaved, trimeric form of the envelope glycoprotein complex of human immunodeficiency virus type 1. J Virol. 2002;76(17):8875–89. PubMed Central PMCID: PMC136973. 12163607 10.1128/JVI.76.17.8875-8889.2002 PMC136973

[39] Wagner S, Dues G, Sawitzky D, Frey P, Christ B. Assessment of the biological performance of the needle-free injector INJEX using the isolated porcine forelimb. Br J Dermatol. 2004;150(3):455–61. 10.1111/j.1365-2133.2004.05853.x 15030327

[40] Palgen J-L, Tchitchek N, Elhmouzi-Younes J, Delandre S, Namet I, Rosenbaum P, et al Prime and Boost Vaccination Elicit a Distinct Innate Myeloid Cell Immune Response. Sci Rep. 2018;8(1):3087 10.1038/s41598-018-21222-2 PubMed Central PMCID: PMC5814452. 29449630 PMC5814452

[41] Vaccari M, Fourati S, Gordon SN, Brown DR, Bissa M, Schifanella L, et al HIV vaccine candidate activation of hypoxia and the inflammasome in CD14+ monocytes is associated with a decreased risk of SIVmac251 acquisition. Nat Med. 2018;24(6):847–56. 10.1038/s41591-018-0025-7 PubMed Central PMCID: PMC5992093. 29785023 PMC5992093

[42] Wood MP, Wood LF, Templeton M, Fisher B, Lippy A, Jones CI, et al Transient Immune Activation in BCG-Vaccinated Infant Rhesus Macaques Is Not Sufficient to Influence Oral Simian Immunodeficiency Virus Infection. J Infect Dis. 2019 10.1093/infdis/jiz382 31605528 PMC7457186

[43] Kaminski DA, Wei C, Qian Y, Rosenberg AF, Sanz I. Advances in human B cell phenotypic profiling. Front Immunol. 2012;3:302 10.3389/fimmu.2012.00302 PubMed Central PMCID: PMC3467643. 23087687 PMC3467643

[44] Tay MZ, Wiehe K, Pollara J. Antibody-Dependent Cellular Phagocytosis in Antiviral Immune Responses. Front Immunol. 2019;10:332 Epub 2019/03/16. 10.3389/fimmu.2019.00332 30873178 PMC6404786

[45] Ackerman ME, Moldt B, Wyatt RT, Dugast AS, McAndrew E, Tsoukas S, et al A robust, high-throughput assay to determine the phagocytic activity of clinical antibody samples. J Immunol Methods. 2011;366(1–2):8–19. Epub 2011/01/05. 10.1016/j.jim.2010.12.016 21192942 PMC3050993

[46] Zolla-Pazner S, Powell R, Yahyaei S, Williams C, Jiang X, Li W, et al Rationally Designed Vaccines Targeting the V2 Region of HIV-1 gp120 Induce a Focused, Cross-Clade-Reactive, Biologically Functional Antibody Response. J Virol. 2016;90(24):10993–1006. Epub 2016/09/16. 10.1128/JVI.01403-16 27630234 PMC5126359

[47] Pugach P, Ozorowski G, Cupo A, Ringe R, Yasmeen A, de Val N, et al A native-like SOSIP.664 trimer based on an HIV-1 subtype B env gene. J Virol. 2015;89(6):3380–95. 10.1128/JVI.03473-14 PubMed Central PMCID: PMC4337520. 25589637 PMC4337520

[48] Klasse PJ, Ketas TJ, Cottrell CA, Ozorowski G, Debnath G, Camara D, et al Epitopes for neutralizing antibodies induced by HIV-1 envelope glycoprotein BG505 SOSIP trimers in rabbits and macaques. PLoS Pathog. 2018;14(2):e1006913 10.1371/journal.ppat.1006913 29474444 PMC5841823

[49] Pauthner M, Havenar-Daughton C, Sok D, Nkolola JP, Bastidas R, Boopathy AV, et al Elicitation of Robust Tier 2 Neutralizing Antibody Responses in Nonhuman Primates by HIV Envelope Trimer Immunization Using Optimized Approaches. Immunity. 2017;46(6):1073–88.e6. 10.1016/j.immuni.2017.05.007 PubMed Central PMCID: PMC5483234. 28636956 PMC5483234

[50] Pauthner MG, Nkolola JP, Havenar-Daughton C, Murrell B, Reiss SM, Bastidas R, et al Vaccine-Induced Protection from Homologous Tier 2 SHIV Challenge in Nonhuman Primates Depends on Serum-Neutralizing Antibody Titers. Immunity. 2019;50(1):241–52.e6. 10.1016/j.immuni.2018.11.011 PubMed Central PMCID: PMC6335502. 30552025 PMC6335502

[51] Torrents de la Peña A, de Taeye SW, Sliepen K, LaBranche CC, Burger JA, Schermer EE, et al Immunogenicity in Rabbits of HIV-1 SOSIP Trimers from Clades A, B, and C, Given Individually, Sequentially, or in Combination. J Virol. 2018;92(8). 10.1128/JVI.01957-17 PubMed Central PMCID: PMC5874403. 29367243 PMC5874403

[52] Binley JM, Sanders RW, Clas B, Schuelke N, Master A, Guo Y, et al A recombinant human immunodeficiency virus type 1 envelope glycoprotein complex stabilized by an intermolecular disulfide bond between the gp120 and gp41 subunits is an antigenic mimic of the trimeric virion-associated structure. J Virol. 2000;74(2):627–43. PubMed Central PMCID: PMC111582. 10623724 10.1128/jvi.74.2.627-643.2000 PMC111582

[53] Klasse PJ, Depetris RS, Pejchal R, Julien J-P, Khayat R, Lee JH, et al Influences on Trimerization and Aggregation of Soluble, Cleaved HIV-1 SOSIP Envelope Glycoprotein. J Virol. 2013;87(17):9873–85. 10.1128/JVI.01226-13 23824824 PMC3754145

[54] Guenaga J, Dubrovskaya V, de Val N, Sharma SK, Carrette B, Ward AB, et al Structure-Guided Redesign Increases the Propensity of HIV Env To Generate Highly Stable Soluble Trimers. J Virol. 2015;90(6):2806–17. 10.1128/JVI.02652-15 PubMed Central PMCID: PMC4810649. 26719252 PMC4810649

[55] Barolet D, Benohanian A. Current trends in needle-free jet injection: an update. Clin Cosmet Investig Dermatol. 2018;11:231–8. 10.2147/CCID.S162724 PubMed Central PMCID: PMC5936486. 29750049 PMC5936486

