# Supplementary figures and images for: Oral Immunization with HIV-1 Envelope SOSIP trimers elicits systemic immune responses and cross-reactive anti-V1V2 antibodies in non-human primates
Source: PLoS One. 2020 May 29;15(5):e0233577. doi: 10.1371/journal.pone.0233577 (PMC7259690; doi:10.1371/journal.pone.0233577)

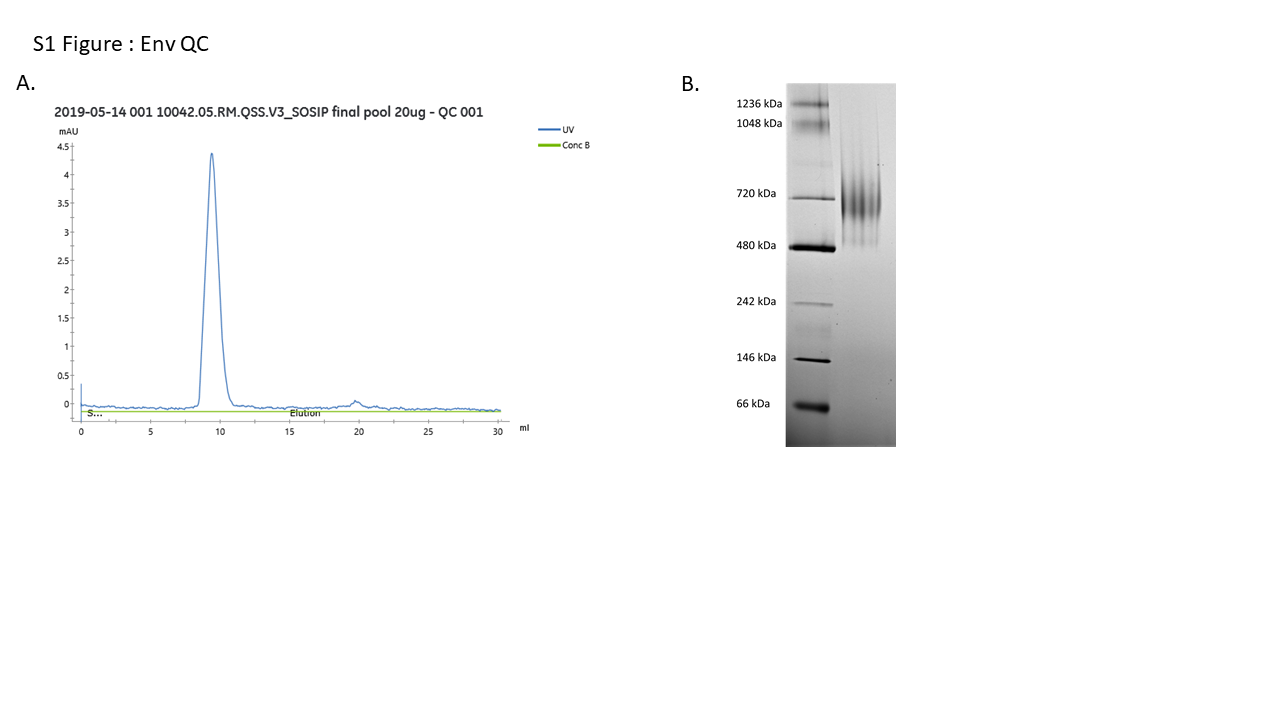

Supplement: S1 Fig — A) Size exclusion chromatography (SEC) profile of 10042.05.SOSIP following lectin affinity purification and ion exchange chromatography. B) Blue native PAGE of final trimer preparation. (TIF) [file pone.0233577.s001.TIF]

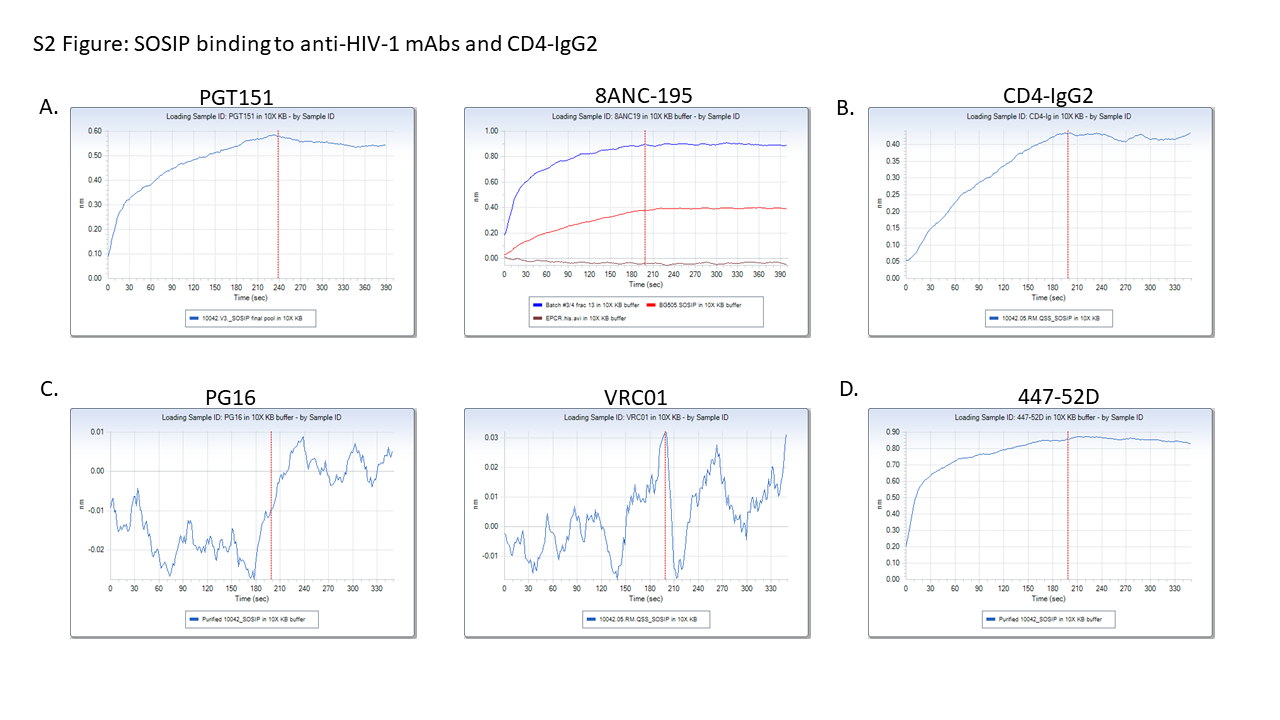

Supplement: S2 Fig — Binding profile of 10042.05.SOSIP to trimer-specific mAbs PGT151and 8ANC195 (A), CD4-IgG2 (B), bNAbs PG16 and VRC01, and the V3-directed mAb 447-52D, as determined by biolayer interferometry(BLI). In (A), human EPCR protein is shown in a red trace as a negative control. (TIF) [file pone.0233577.s002.TIF]

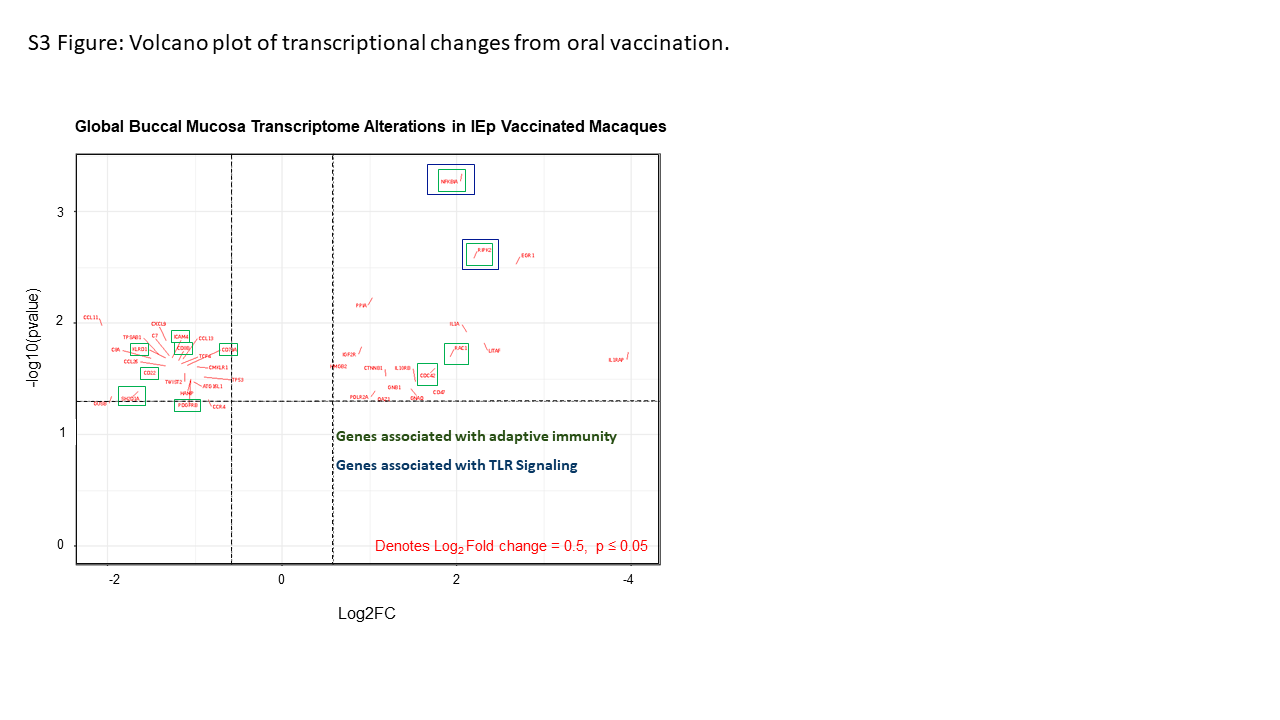

Supplement: S3 Fig — Oral buccal cytobrushes were collected week 1 following the first immunization in both oral iEp vaccinated macaques and in systemic IM vaccinated macaques. Differentially expressed genes were determined by comparing the gene signature in iEp immunization to IM immunization. Global transcriptome volcano plot showing genes with a 0.5 log2 fold change and p ≤ 0.05 in iEp immunized macaques compared IM immunized macaques denoted in red. Statistical significance was determined by t-tests. (TIF) [file pone.0233577.s003.TIF]

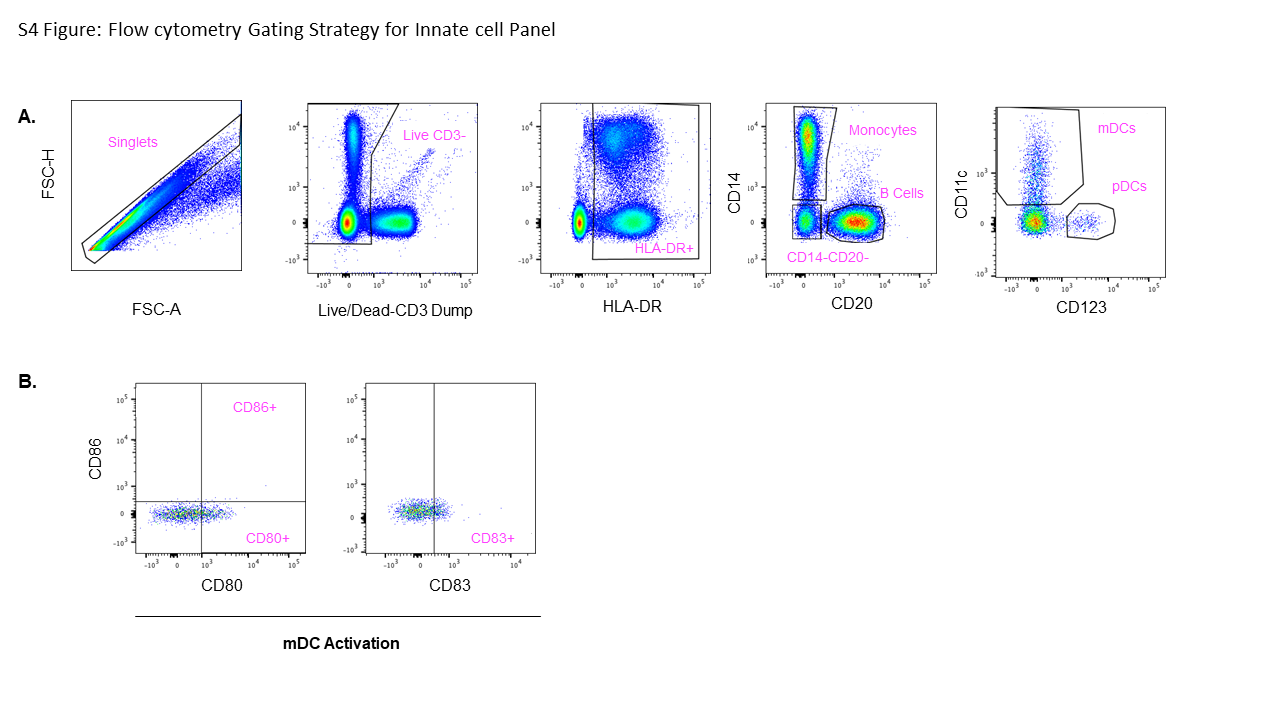

Supplement: S4 Fig — A) Stained PBMCs were first gated on single, live, CD3- cells, followed by identification of myeloid dendritic cells (mDCs) using the markers HLADR+CD14-CD20-CD11c+. B) mDCs were then phenotyped using the markers CD80, CD86 and CD83. (TIF) [file pone.0233577.s004.TIF]

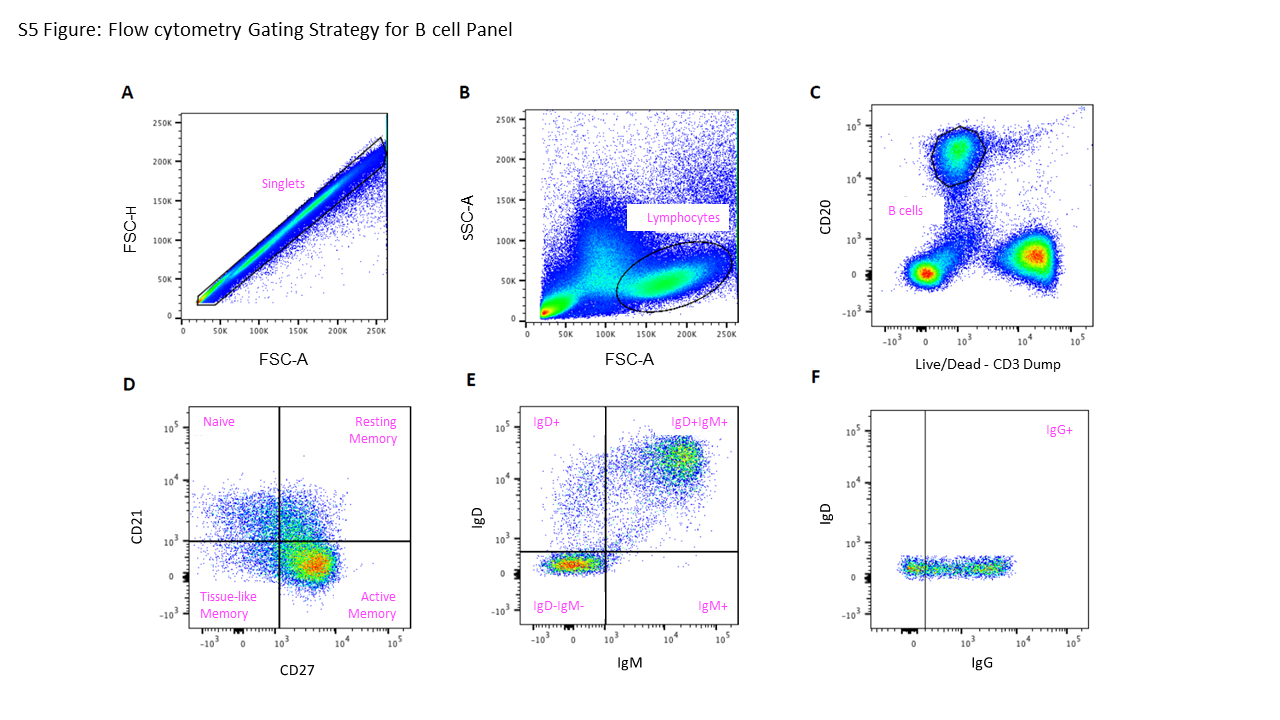

Supplement: S5 Fig — The gating strategy used to define the B cell populations in the peripheral blood consisted of gating on singlets (A), then lymphocytes (B), followed by exclusion of dead/CD3+ cells (C). The surface markers CD21 and CD27 were used to distinguish the following B cell subsets: activated memory (CD20+CD21-CD27+), resting memory (CD20+CD21+CD27+), tissue-like memory (CD20+CD21-CD27-) and naive (CD20+CD21+CD27-) (D). The expression of surface immunoglobulin M (IgM) and D (IgD) within each B cell subset was determined as shown (E). The expression of surface immunoglobulin G (IgG) was determined by first gating on the IgD-IgM- population, followed by gating on the IgG+ population (F). (TIF) [file pone.0233577.s005.TIF]

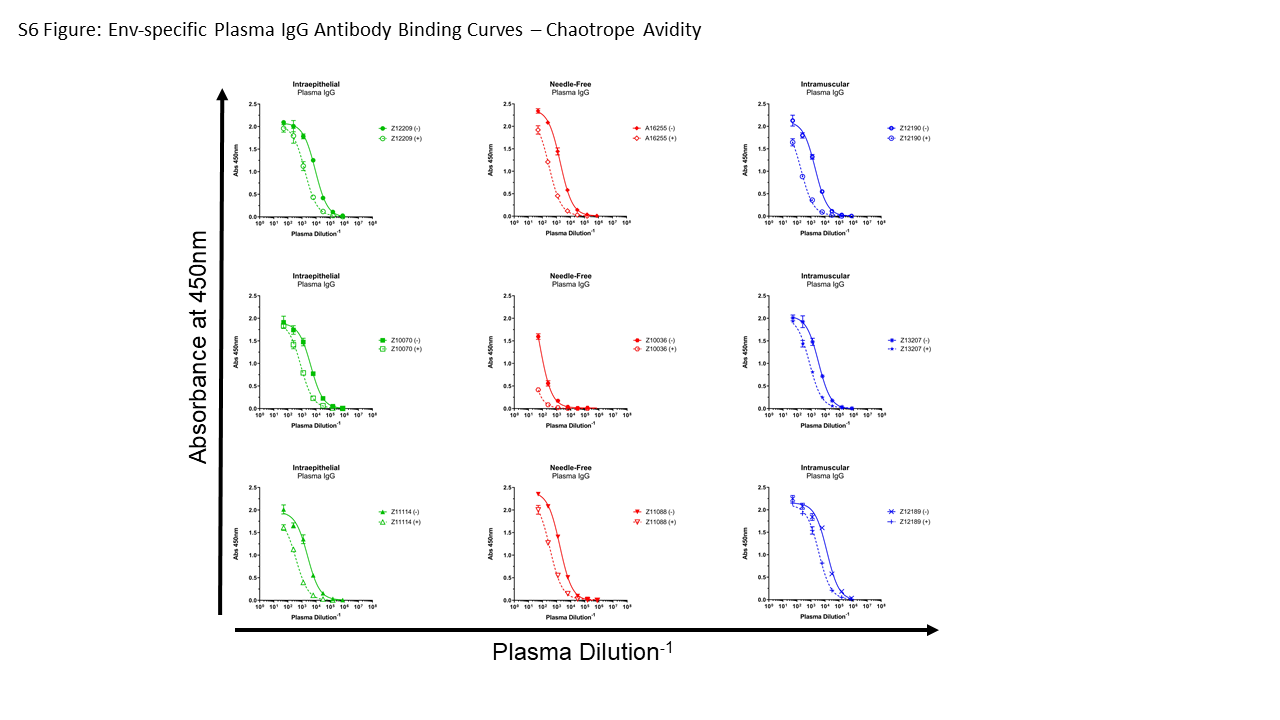

Supplement: S6 Fig — Avidity of envelope-specific IgG (week 17) was measured by ELISA using 2M ammonium thiocyanate (NH4SCN) treatment. Individual macaques are denoted by symbol color and shape, NH4SCN-treated samples are indicated by dashed lines, PBS-treated samples are indicated by solid lines. (TIF) [file pone.0233577.s006.TIF]

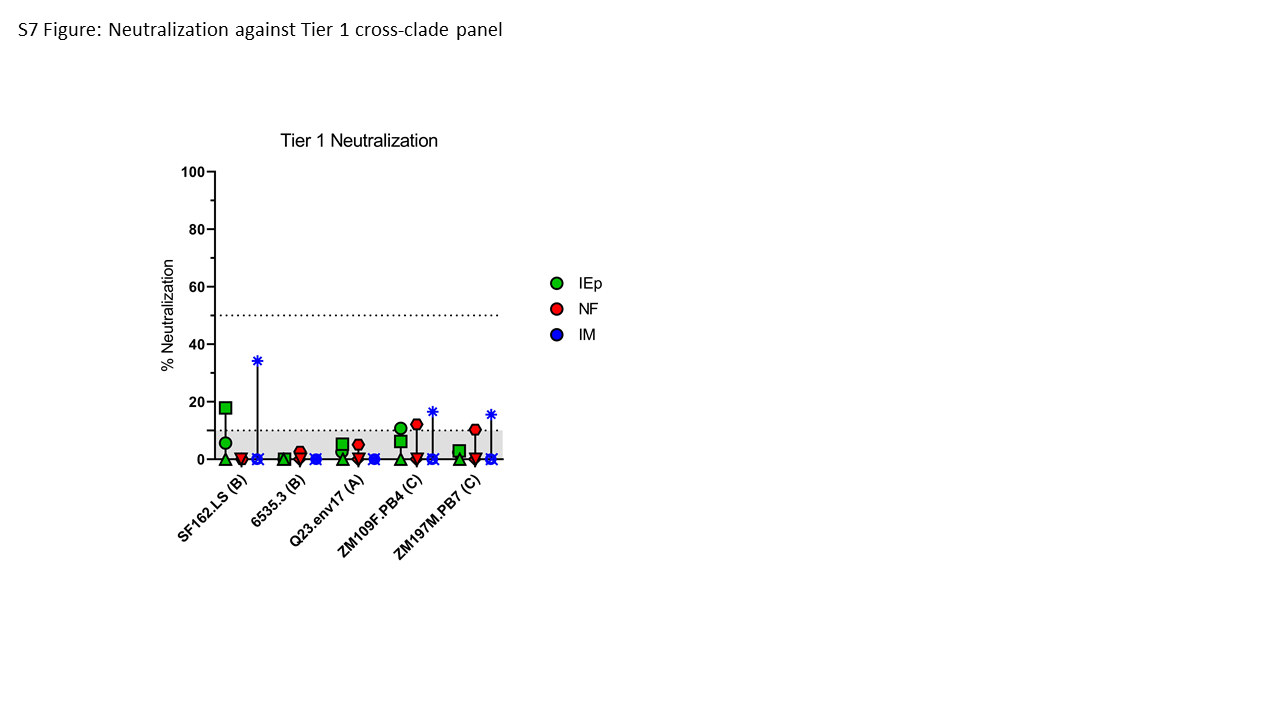

Supplement: S7 Fig — Plasma from week 17 (1 week post 3rd immunization) were tested for neutralizing activity in the TZM-bl assay. Plasma were tested a dilution of 1:50 in triplicate wells and compared against virus-alone entry. Each data point represents the average of triplicate wells. The viruses derive from clades A, B, and C, and are known to have a tier 1, easy to neutralize phenotype. The standard cutoff of 50% is noted by a dotted line. (TIF) [file pone.0233577.s007.TIF]
